# Unraveling the Phytochemistry, Traditional Uses, and Biological and Pharmacological Activities of *Thymus algeriensis* Boiss. & Reut

**DOI:** 10.1155/2022/6487430

**Published:** 2022-05-25

**Authors:** Ismail Mahdi, Widad Ben Bakrim, Gabin Thierry M. Bitchagno, Hassan Annaz, Mona F. Mahmoud, Mansour Sobeh

**Affiliations:** ^1^Agrobiosciences, Mohamed IV Polytechnic University, Lot 660, Hay Moulay Rachid, Ben Guerir 43150, Morocco; ^2^African Sustainable Agriculture Research Institute (ASARI), Mohammed VI Polytechnic University (UM6P), Laayoune, Morocco; ^3^Department of Pharmacology and Toxicology, Faculty of Pharmacy, Zagazig University, Zagazig 44519, Egypt

## Abstract

Growing concern for public health has increased the need to change the paradigm towards a healthcare system that advocates holistic practices while reducing adverse effects. Herbal therapy is becoming an integral part of the therapeutic arsenal, and several successful plant-derived compounds/molecules are being introduced into the market. The medicinal plants belonging to the genus *Thymus* are among the most important species within the Lamiaceae family. One of them is *Thymus algeriensis* which is mainly distributed in the Mediterranean region. For a long time, this species has been used in traditional medicine to treat several disorders and diseases including inflammation, diabetes, rheumatism, digestive, and respiratory affections. This review describes the traditional uses, phytochemical composition, and biological and pharmacological activities of *T. algeriensis* extracts. Data were obtained using electronic databases such as SciFinder^n^, ScienceDirect, Scopus, and Web of Science. Several plant-based extracts and a broad spectrum of identified secondary metabolites were highlighted and discussed with respective activities and modes of action. *T. algeriensis* represents a promising natural resource for the pharmaceutical industry mainly for antioxidant, anti-inflammatory, antimicrobial, and anticancer activities. Considering these findings, more research is needed to transmute the conventional uses of *T. algeriensis* into scientifically sound information. Moreover, extensive preclinical, clinical, toxicological, and pharmacokinetic trials on this species and its derivatives compounds are required to underpin the mechanisms of action and ensure its biosafety and efficiency. This comprehensive review provides a scientific basis for future investigations on the use of *T. algeriensis* and derived compounds in health maintenance and promotion and disease prevention.

## 1. Introduction

According to the World Health Organization (WHO), about 80% of the earth's population relies on folk medicine. Most of ethnopharmacological practices involve the use of plant-based extracts and their bioactive constituents as natural healing remedies [1]. Plants have been used for therapeutic purposes worldwide for thousands of years and still provide the largest drugs to humankind. Therefore, scientists have dedicated a lot of effort to drug discovery willing to identify natural molecules/compounds from plants [2]. Until now, the international market of medicinal and aromatic plants has reached over 60 billion dollars per year, and it is still increasing gradually [3]. In addition, the pharmaceutical industry values medicinal plants for their bioactive constituents such as flavonoids, polyphenols, alkaloids, tannins, and glycosides, which are used as agents in drugs' synthesis [4]. Nowadays, plant-derived molecules are continuously enriching our drug arsenal (e.g., galantamine, vinblastine, vincristine, and artemisinin) [5].

*Thymus* is a large plant genus comprising up to 400 species of aromatic and medicinal herbaceous, perennials, and shrubs. They are widely distributed in the Mediterranean region and Asia. *Thymus* species are used traditionally as herbal teas, culinary spices, and condiments [6]. Additionally, their essential oils are listed as one of the world's top ten essential oils and known for their broad spectrum of biological activities including antioxidant, antibacterial, and age-delaying properties [7].

One of the most renowned North African *Thymus* species is *T. algeriensis*. The previous investigations carried out on this plant species have been mainly oriented towards its biological activities and clinical attributes. According to the Scopus database, more than 43.4% of the works relate to biological, pharmacological, biochemical, microbiological, and immunological aspects. The largest number of these studies was carried out by North African researchers and institutions, particularly Algerian, Moroccan, and Tunisian. In the Scopus database, the TITLE-ABS-KEY (*thymus* AND *algeriensis*) research resulted in 951 documents gathering articles (95.5%), reviews (2.2%), book chapters, and data papers (2.2%) with an increasing trend of document numbers over the years. For instance, it went from 4 to 15 documents per year between 2013 and 2021 indicating the importance that this species arouses among researchers. Therefore, *T. algeriensis* is a promising endemic resource for drug discovery and healthcare systems. Since this *Thymus* species has been the subject of a multitude of studies using both *in vitro* and *in vivo* approaches, and the data related to its phytochemistry and biological properties are distributed in several documents, we thought, here, to comprehensively summarize and review the phytochemical composition of *T. algeriensis* tissues with reference to the biological and pharmacological activities of its various extracts to have a holistic and synoptic view of its benefits and curative potentialities and track down research gaps and future prospects.

## 2. Botanical Description and Distribution

**Figure 1:**
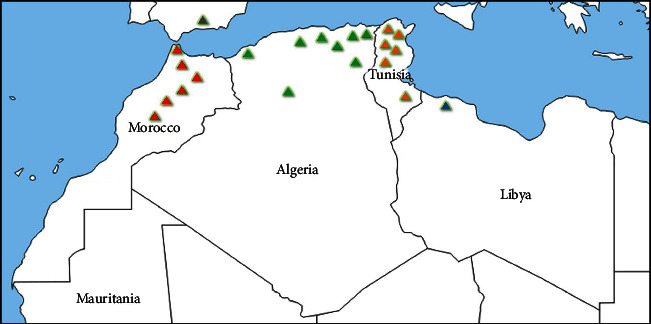
Map of distribution of *T. algeriensis* Boiss. & Reut. across North Africa.

*T. algeriensis* is an endemic species of North Africa (Morocco, Algeria, Tunisia, and Libya) (Figure 1) [8]. The *Thymus* genus is represented by numerous aromatic plant species, including *T. algeriensis* which is a short lived, diploid (2*n* = 2*x* = 30) and gynodioecious shrub [9, 10] belonging to the *Hyphodromi* section and the *Subbracteati* subsection [11]. It grows wildly in diverse bioclimatic areas extending from the subhumid to the lower arid and on poor fertile calcareous soils [12]. In Morocco, it is found in the Middle, the High, and the Western Anti-Atlas, the Rif, and the Oriental (Figure 1).

**Figure 2:**
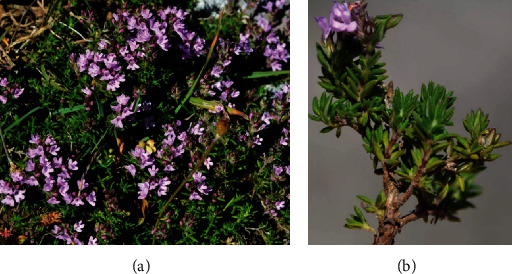
(a) *Thymus algeriensis* Boiss. & Reut. plant; (b) aerial part of *Thymus algeriensis* Boiss. & Reut. (Source: https://www.biodiversidadvirtual.org/).

*T. algeriensis* is a perennial plant with 4 to 7 mm long internodes emerging as a tuft from the short woody stump [13] (Figure 2(a)). The leaves are opposite with linear-lanceolate (6–12 mm) and have both green surfaces. The flowers are small (5 –7 mm) and have a white purplish or pinkish purple corolla color, with the upper lip cleft and the lower one divided into three lopes (Figure 2(b)). Flowering and fruiting time takes place from late April to June [14, 15].

Due to the anthropic pressures (overcollection, overgrazing, clearing, etc.), *Thymus* populations and cultivars from natural areas are severely affected and tend to occur in scattered metapopulations, often characterized by a low size [9]. Many factors influence the level of differentiation and the genetic drift of *T. algeriensis* populations, mainly habitat fragmentation, special isolation, ecological conditions, and gene flow limitation reducing their adaptation to ecological changes [16]. Several chemotypes were described in *T. algeriensis* according to their phytochemical composition (essential oils and main compounds) [15]. The genetic diversity among populations are also reported to be influenced by the level of site destruction, the number of initial founders in populations, and their dispersal and reproductive potentials [16].

## 3. Sources, Search Strategy, and Eligibility Criteria

The chemical composition and the biological and pharmacological activities of *T. algeriensis* were obtained using the electronic databases SciFinderⁿ®, Web of Science, Google Scholar, and Scopus. The search term used was *Thymus algeriensis* Boiss. & Reut. (1210 records). When crosslinked with specified terms, mainly “chemical compounds” (1010 records) and “activities” (1050 records), the number of documents decreased to 989. The cumulative results were then crosslinked with biological activities, *in vitro*, *in vivo*, or pharmacology which resulted in 155 records. In addition, titles and abstracts were screened and subjected to inclusion criteria that were as follows: phytochemical constituents of *Thymus algeriensis* extracts and their biological and pharmacological activities both *in vitro* and *in vivo*. Exclusion criteria were also considered and included other applications of the plant such as agriculture, non-English documents, duplicated papers, and the inability to locate full text. This selection resulted in relevant literature of 87 records that was used to retrieve data represented in this review. Other than the aforementioned records, many other references were sourced from citations of eligible studies.

## 4. Phytochemical Composition of *T. algeriensis*

**Figure 3:**
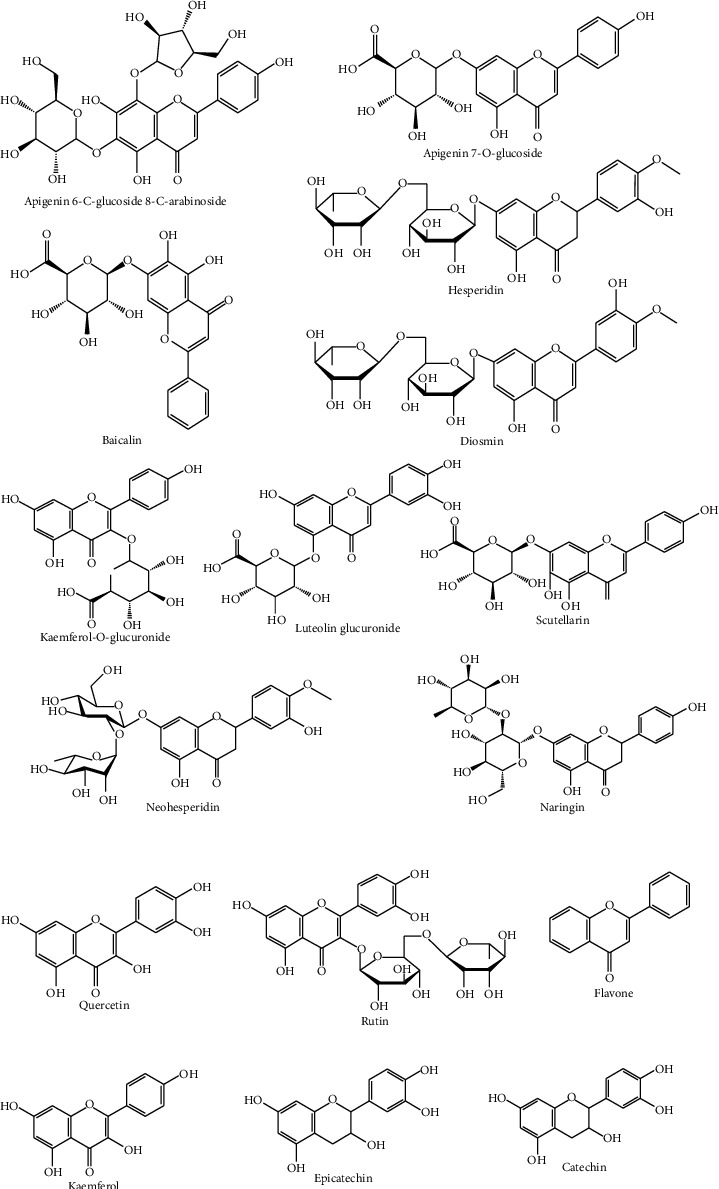
Flavonoids identified from *T. algeriensis*.

The phytochemical analysis of different parts of *T. algeriensis* has shown the presence of diverse phytochemicals like polyphenols, flavonoids, terpenoids, sterols, and volatile compounds. This might be attributed to different factors such as geographic location, temperature, and harvesting time. Furthermore, phytochemical content is reported to vary with the extraction method employed and compounds identified in various parts of *T. algeriensis*. The major active constituents are flavonoids (Figure 3). The plant is also known by the volatile compounds characterizing the essential oil. Tables 1 and 2 demonstrate the reported compounds from *T. algeriensis* of different parts including aerial parts, leaves, flowers, and stem bark. The presence of various phytochemicals in *T. algeriensis* suggests its pluripharmacological properties, and a comprehensive assessment of the various activities of different phytochemicals is included in the sections below.

### 4.1. Aerial Parts

Most of the studies have been focused on the aerial parts of *T. algeriensis*. Different studies reported that the hydroalcoholic extracts were shown to contain kaempferol-*O*-glucuronide, apigenin-6,8-*C*-dihexoside and apigenin-7-*O*-glucuronide, and naringenin, identified from the Algerian plants [7, 17, 18], while apigenin was annotated in the polar extracts of the Tunisian plants [14, 19] (Figure 3). In the Tunisian plants, catechin, epicatechin, rutin, flavone, and (+)-catechin hydrate have been identified in the hydroalcoholic and methanolic extracts (Figure 3) [14, 18, 19].

**Figure 4:**
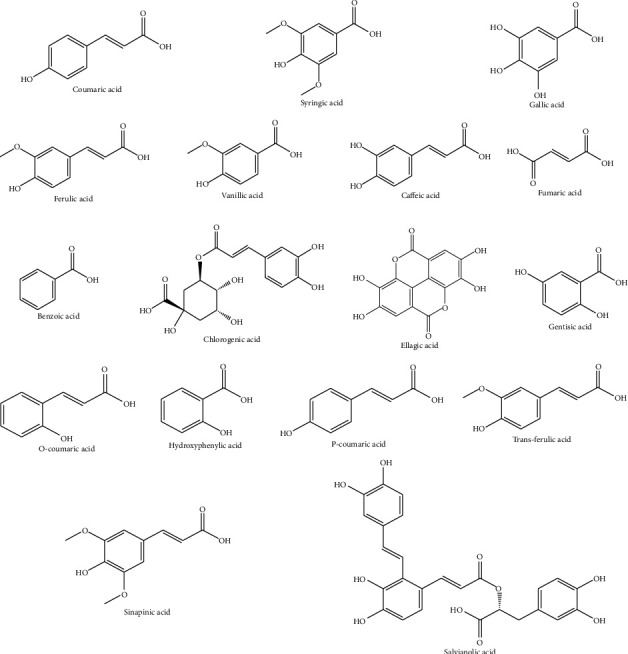
Phenolic and carboxylic acids identified from *T. algeriensis*.

**Figure 5:**
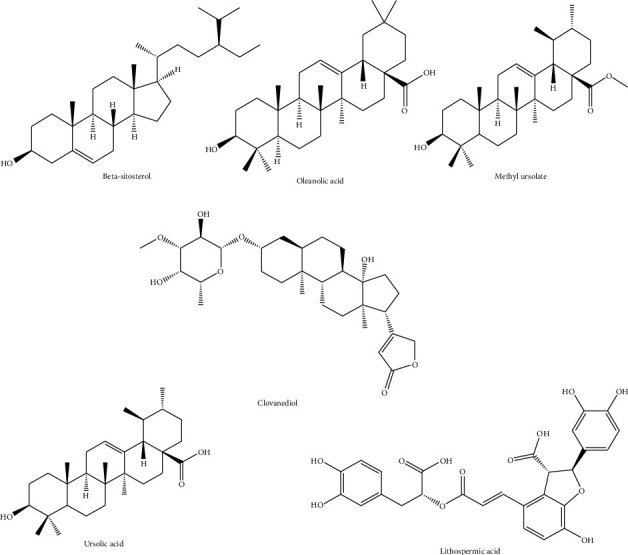
Other compounds identified from *T. algeriensis*.

Phenolic acids such as *O*-coumaric, *p*-coumaric, salvianolic, and t-ferulic were documented from the Algerian flora [7, 18], while gallic, rosmarinic, syringic, and vanillic acids were detected in the extracts from the Tunisian and Egyptian plants (Figure 4). In contrast, caffeic and ferulic acids characterized the methanolic extract of the plant harvested from Tunisia [20]. In the same line, 2,3-dimethoxybenzoic acid, 3-hydroxybenzoic acid, and 4-hydroxybenzoic acid were identified in the ethanol, hydroalcoholic, and butanolic extracts of the Algerian plants (Figure 5) [18].

### 4.2. Leaves

*T. algeriensis* has a high antioxidant activity mainly due to its high content in flavonoids (Figure 3). Most of the flavonoids present in the leaves are in the flavanol and glycoside forms. Kaempferol and rutin were detected in the polar leaves' extract of the plants from Algeria and Tunisia. Kaempferol-*O*-hexoside and kaempferol-*O*-hexuronide have been identified in the methanolic extract of the leaves collected from Tunisia [21], while luteolin glucuronide was identified in the Algerian plants (Figure 3) [22]. From all phenolic acids (Figure 4), the leaves of *T. algeriensis* are rich in vanillic and rosmarinic acids, well-known phenolic acids with previously confirmed biological and pharmacological activities. These two compounds were characterized in the Algerian and Tunisian plants [7, 18–21, 23, 24]. The plant also contained phenolic acid derivatives such as rosmarinic acid glucoside, characterized in the methanolic extract of the Algerian plants [22]. Ursolic acid, a triterpenoid, was identified in the methanolic extract of the leaves. In addition, sterols such as *β*-sitosterol have been identified in the methanolic extract of the leaves. In addition, oleanolic acid, a triterpenoid, was identified in the leaves [25] (Figure 5).

### 4.3. Essential Oils of *T. algeriensis*

**Figure 6:**
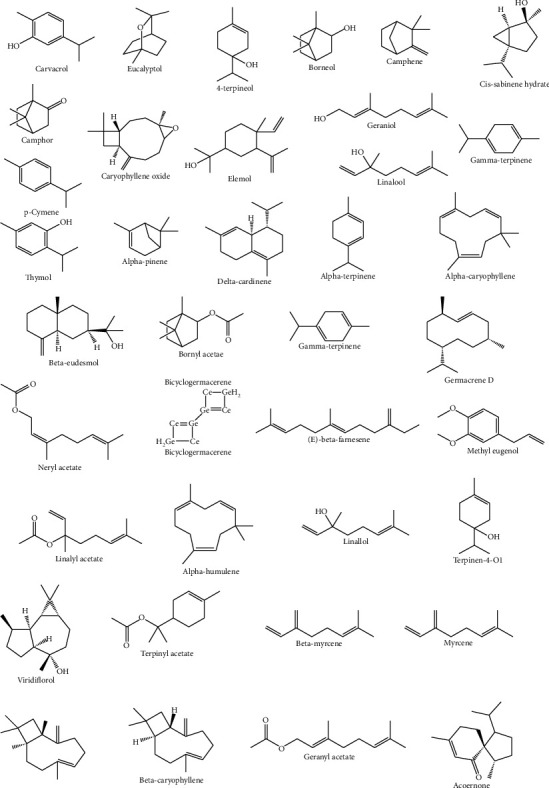
Selected volatile compounds identified from *T. algeriensis*.

Essential oils are naturally defined as volatile secondary metabolites of plants and characterized by a strong aromatic nature and a complex chemical composition. The aroma of *T. algeriensis* is strong and contains large quantities of volatile compounds [26, 27]. More than 40 volatile compounds have been identified in *T. algeriensis* (Table 2 and Figure 6).

Several investigations reported the phytochemical composition of the essential oils of different specimen of *T. algeriensis* from different regions including Algeria, Libya, Tunisia, and Morocco (Table 2). The monoterpenoids and sesquiterpenoids dominated the oil. For instance, the main components of the essential oils of the aerial parts from the Tunisian flora were 1,8-cineole and 4-terpineol [9, 15], while borneol, camphene, camphor, and carvacrol dominated the Algerian and Moroccan plants. *p*-Cymene and thymol were detected in all flora. In the Tunisian flora, caryophyllene oxide and cis-sabinene hydrate were identified from different parts of the plant with different percentages [15, 28, 29]. Limited studies focused on the isolation and identification of bioactive compounds from the flowers of *T. algeriensis*. Like the leaves, the flowers were shown to contain 1,8-cineole identified in the Algerian plants. Elemol was identified from the stem bark from the Tunisian plants while acorenone and *α*-pinene were characterized in the stem bark of the Algerian plants [23, 30].

The variations in the phytochemical composition of the essential oils could be attributed to the harvesting time and stage and the drying methods as well as extraction methods. Some factors like environmental conditions, genetic variations, physiological condition of plants, regions, and evolution also influence the phytochemical variability of *T. algeriensis* [31]. Overall, *T. algeriensis* alongside other Lamiaceae species contains high contents of polyphenols, flavonoids, terpenoids, sterols, and volatile compounds.

## 5. Traditional Uses and Ethnomedicinal Properties

*Thymus* species have been used by the populations of the Mediterranean and Asian countries for thousands of years [32, 33]. Traditional health uses of *Thymus* species show high applicability as flu controller, anti-inflammatory, sedative, antirheumatic, analgesic, antiseptic, astringent, and diuretic agents. Generally, *Thymus* flowers and leaves are mainly used as herbal teas and aromatic and flavoring preparations to treat common cold, cough, sore throat, and indigestion symptoms [33]. *Thymus* leaves are also used as astringent, expectorant, antiseptic, antirheumatic, diuretic, analgesic, and cicatrizing agents [18]. *T. algeriensis* is also used either fresh or dried for its antispasmodic, antiabortive, and antifungal properties [34, 35]. In Morocco, its vernacular name is either Zîtra, Tazouknit, or Mantha [36]. Infusion and decoction of its stems and/or leaves are traditionally used to treat diabetes and digestive and respiratory infections [37, 38]. To sum up, *T. algeriensis* preparations are traditionally known for their multiple benefits and uses in gastronomy, digestive and cold problems, analgesia, microbial infections, and perfume preparations.

## 6. In Vitro Pharmacological Properties

Many studies have shown that *T. algeriensis* extracts have several *in vitro* biological properties mainly antioxidant, anti-inflammatory, antimicrobial, and anticancer activities.

### 6.1. In Vitro Antioxidant Activity

The oxidation process causes cellular damage by interacting with biological materials within the cell leading to several disorders and chronic diseases such as cardiovascular diseases and cancer. In addition, oxidation forms secondary reaction products in food and alters its nutritional quality and safety [4].

Antioxidant activity was defined as a delay or inhibition of the oxidation of cell molecules mainly proteins, lipids, DNA, and sugars by limiting the oxidative chain reactions. Due to their phytochemicals, plants are generally known as the best source of active antioxidants [39]. This is mainly attributed to their capacity to prevent the oxidation of a substrate by neutralizing reactive oxygen species (ROS) such as superoxide radical and hydroxyl radical. Common mechanisms by which plant-based extracts/molecules block ROS formation include mainly free radical scavenging (e.g., lipoxygenase inhibition, transition-metal-chelating activity, and singlet-oxygen-quenching capacity) and lipid peroxidation inhibition [40, 41]. Hence, there is a growing interest in the antioxidant activities of plant-based compounds and their role in promoting health and preventing diseases.

The *in vitro* antioxidant activities of *T. algeriensis* extracts have been extensively explored (Table 3). They have been determined by various methods mainly 2,2-diphenyl-1-picrylhydrazil (DPPH) radical scavenging, ferric-reducing antioxidant power (FRAP), *β*–carotene bleaching, oxygen radical absorbance capacity (ORAC), thiobarbituric acid reactive substances (TBARS), reducing power (RP), phosphomolybdenum, lipid peroxidation inhibition, total antioxidant capacity (TAC), hydroxyl radical scavenging (HRS), metal ion chelation, and superoxide anion scavenging assays (Table 3).

Many studies have been carried out on the *T. algeriensis* aerial parts using mainly EO. However, other extracts such as methanolic, aqueous, and ethanolic were also tested (Table 3). Recently, Ouakouak et al. [42] showed that the EO from the leaves of Algerian *T. algeriensis* are endowed with a moderate antioxidant activity using DPPH (IC_50_ (mg/mL) = 41.09), ABTS (IC_50_ (mg/m) = 10.84), and TAC (TAC (U/L) = 39.27 ± 3.47) assays. This activity was corroborated in other studies that showed that the EO extracted from the aerial parts of *T. algeriensis* grown in Libya possessed a strong antioxidant activity (IC_50_ = 0.299 mg/mL) better than thymol [43] and inhibited the deoxyribose degradation better than mannitol [35]. The only study on the antioxidant potential of EO from *T. algeriensis* stem bark was conducted in Algeria and showed that the plant exhibited moderate DPPH scavenging activity (IC_50_ = 83.8 mg/mL) [30]. Furthermore, Mokhtari et al. [44] tested the antioxidant activities of other different extracts and showed that, using the DPPH assay, chloroform, petroleum ether, and n–BuOH extracts demonstrated IC_50_ values of 79.92 ± 0.30, 69.50 ± 0.68, and 5.05 ± 0.12 *μ*g/mL, respectively. Noteworthy, the antioxidant potential of all these extracts was dependent on the method used. For instance, using the CUPRAC assay, the chloroform extract was the most effective while the n–BuOH extract was the most active using the FTC assay. In another work, the aqueous extract of leaves of Algerian *T. algeriensis* was active only using ABTS (IC_50_ = 52 ± 31 *μ*g/mL) while the ethanolic extract was active using both the DPPH and ABTS assays with IC_50_ (*μ*g/mL) of 52 ± 4 and 42 ± 0.99, respectively [45]. In contrast, Ziani et al. showed that both aqueous and hydroethanolic extracts exhibit antioxidant activities using DPPH, RP, *β*–carotene bleaching, and TBARS assays, with a high efficiency of the hydroethanolic fraction except when using *β*–carotene bleaching in which the aqueous extract demonstrated the highest activity [7]. It was also shown that the ethyl acetate extract is also endowed with an antioxidant capacity (IC_50_ = 0.290 mg/mL) higher than the n–BuOH extract (IC_50_ = 1.45 mg/mL) and ascorbic acid [46]. Interestingly, Rezq et al. [47] demonstrated that methanolic extracts of *T. algeriensis* leaves induced significant increase in the nuclear levels of Nrf–2 (nuclear factor erythroid 2) transcription factor (Table 3). The Nrf–2 upregulates the antioxidant response element-mediated expression of antioxidant enzymes and cytoprotective proteins and protects against oxidative pulmonary injury, abnormal inflammatory and immune responses, and apoptosis [48]. The various antioxidant activities of *T. algeriensis* extracts were largely studied and correlated with their phytochemical composition. In this regard, it has been shown that the Algerian *T. algeriensis* EO with poor content of thymol and carvacrol exhibited significantly lower antioxidant effect [35]. Similarly, Guesmi et al. [20] concluded that the higher the polarity of the Tunisian *T. algeriensis* extract, the stronger is the antioxidant effect. In addition, the antioxidant effect was chemotype dependent [20]. This was also reported by Jaouadi et al. [21] who showed that *T. algeriensis* plants growing in upper arid climatic zones of Tunisia were endowed with the best reducing, antiradical, and *β*–carotene bleaching inhibition potential. Likewise, the antioxidant effect of EO from leaves was reported to vary according to localities [28]. Furthermore, the antioxidant capacity was also reported to be influenced by the development stage of the plant. For instance, the aqueous extract extracted during the flowering stages of Tunisian *T. algeriensis* had the highest scavenging potential, while the extracts of both vegetative and flowering periods were able to reduce Fe^3+^ to Fe^2+^ [14].

Several studies have compared *Thymus* species to other plants in terms of their antioxidant activities. For instance, Ahmed et al. observed that EO of *T. algeriensis* from Tunisia presented a moderate DPPH scavenging effects compared to EO from other plants such as *Eucalyptus globulus*, *Pinus halepensis*, *Pituranthos tortuosus*, *Rosmarinus officinalis*, and *Tetraclinis articulata* [49]. Similarly, the EO of Moroccan *T. algeriensis* had a lower antioxidant activity comparatively to other species mainly *T. capitatus*, *T. ciliatus*, and *T. bleicherianus* [50]. However, because of the bioassay used, it is often biased and difficult to compare the antioxidant activities reported by many different studies [51].

The antioxidant effect of *T. algeriensis* extracts and EO is attributed to their free radical scavenging and metal chelating properties having a chemoprotective activity against oxidative stress [35]. This should be due to the richness of *T. algeriensis* in phytochemical active compounds dominated by certain phenolic constituents as well as the synergetic effect between its compounds even at small concentrations such as *δ*-cadinene and germacrene D [52]. Next to *T. algeriensis*, other *Thymus* species such as*T. vulgaris*, *T. serpyllum*, *T. bleicherianus*, and *T. hyemalis* have been widely explored for their antioxidant potential [50, 53–56]. As a result, the antioxidant potential has diverse downstream biological effects, including anti-inflammatory, anticarcinogenic, and antiatherosclerosis activities [57]. Same as other Lamiaceae species as well as most medicinal plants, *T. algeriensis* can serve as a good source for antioxidant compounds/molecules with potential applications in pharmaceutical, nutraceutical, and cosmeceutical industries.

### 6.2. Anti-Inflammatory Activity

Inflammation is a healing process induced by inflammation-inducing factors such as pathogens, toxic substances, and irradiation. These stimuli trigger the immune system and induce inflammatory responses in the host's organs which may lead to cell damage and/or diseases [58]. Many diseases are associated with inflammatory processes including type 2 diabetes, asthma, rheumatoid arthritis, neurodegenerative diseases, chronic inflammatory bowel diseases, and cancer [59]. Prominent anti-inflammatory molecules have been isolated from plants and clinically tested in humans. They mainly include curcumin, resveratrol, colchicine, quercetin, capsaicin, and epigallocatechin-3-gallate [5]. The *in vitro* anti-inflammatory effect of *T. algeriensis* has been reported by many studies. It was evaluated using egg albumin denaturation, cyclooxygenase (COX–1 and COX–2), and lipoxygenase inhibition assays (Table 4).

Recently, Mokhtari et al. [44] showed that petroleum ether, chloroform, and n–BuOH extracts from the aerial parts of Algerian *T. algeriensis* inhibited egg albumin denaturation in a concentration-dependent effect. The highest inhibitory effect was observed using the chloroform extract with 45.27% inhibition, followed by petroleum ether (30.26%) and then n–BuOH (26.03%) extracts [44]. This activity could be useful in inhibiting protein denaturation during inflammatory conditions characterized by the production of autoantigens during disorder conditions such as rheumatic arthritis, cancer, and diabetes [60]. As previously suggested, the *in vitro* antidenaturation of protein activity of these extracts could be due to the interactions of the phytocompounds and/or molecules such as polyphenols, phenylpropanoids, and the alpha-lipoic acid with the aliphatic regions surrounding the lysine residue of the albumin protein [61].

Additionally, it was revealed that the methanolic extract of *T. algeriensis* leaves showed higher selectivity against cyclooxygenase (COX–2) (IC_50_ = 0.05 ± 0.01 *μ*M) than both diclofenac and indomethacin used as standard references. Moreover, *T. algeriensis* extract induced similar potency like zileuton and diclofenac to inhibit 5–lipoxygenase (5–LOX) *in vitro* (IC_50_ = 2.70 ± 0.23 *μ*M) [22]. Similarly, the EO of the aerial parts from *T. algeriensis* grown in Algeria were the most potent in inhibiting the lipoxygenase comparatively to those extracted from *Mentha spicata* and *Ocimum basilicum* [62]. This could be associated with the volatile compounds in EO such as 1,8-cineole, borneol, and camphor which were reported to be determinant for the anti-inflammatory activity [63]. For instance, carnosol and rosemary oil inhibited the adhesion of TNF-*α*-induced monocytes to endothelial cells during inflammation and suppressed the expression of intercellular adhesion molecule at the transcriptional scale [64]. Using molecular docking of some *T. algeriensis* compounds characterized in the methanolic extract of leaves, Ref. [22] demonstrated that salvianolic acid A and apigenin 6,8-di-*C*-hexosides interact with amino acid residues of COX–1 and 5–LOX, while rosmarinic acid glucoside interacts with amino acid residues of COX–2. In addition, salvianolic acid A and quercetin pentoside target the amino acid residues of the 5–LOX activating protein (FLAP). This in silico study showed that identified compounds especially rosmarinic acid glucoside, salvianolic acid A, quercetin pentoside, and apigenin 6,8-di-*C*-hexosides could be developed as safer anti-inflammatory agents. Noteworthy, the anti-inflammatory potential of *T. algeriensis* species growing in other North African countries, namely, Morocco, Tunisia, and Libya, has not been evaluated yet (Table 4). In conclusion, *T. algeriensis* is endowed with an exceptional anti-inflammatory power thanks to its compounds and active molecules that target key stages and mediators of the inflammatory process of different pathologies.

### 6.3. Antimicrobial Activities

The overuse of antibiotics has become one of the greatest challenges in human health. In addition, the rapid spread of antibiotic resistant pathogens is alarming. Therefore, research and development of a new generation of antimicrobials has become imperative. Antimicrobial agents are a group of materials that selectively destroy pathogens by interfering with their growth or survival. With the emergence of resistance phenomenon to current antibiotics, new alternative compounds such as plant-derived compounds are being explored. Due to the advantages of their inherent biochemical and biophysical properties including biocompatibility, biodegradability, and low cytotoxicity, plant biomolecules have huge potential for the antimicrobial application and have been extensively studied in recent years.

Several methods are used to evaluate the antimicrobial activities of plant extracts mainly agar disk diffusion, agar well diffusion, and macrodilution or microdilution methods [65]. These methods determine the inhibition zone diameter and the minimal inhibitory (MIC) and bactericidal concentrations (MBC). The antimicrobial activities of the *T. algeriensis* extracts and EO against a broad set of pathogenic bacteria, fungi, and yeasts were exhaustively studied (Tables 5 and 6).

According to literature, EO were the most explored for the antimicrobial potential. For instance, Ait-Ouazzou et al. tested the effect of the EO from the aerial parts of *T. algeriensis* growing in Morocco against seven bacteria and one fungus and showed their very high effectiveness (MIC ≤ 0.5 *μ*L/mL) towards *S. aureus, E. faecalis, L. monocytogenes*, and *C. glabrata* and a moderate activity (MIC = 1.0 *μ*L/mL) against *P. aeruginosa*, *S. enteritidis*, and *E. coli* O157:H7 [66]. These bacteria especially *P. aeruginosa* were sometimes shown to exhibit a remarkable resistance against OE of *T. algeriensis* from other countries such as Tunisia and Algeria [20, 28, 67]. Interestingly, Hazzit et al. [35] noticed that *Helicobacter pylori* strains J99 and 26695 were more sensitive (IZ = up to 30 mm) to EO from Algerian *T. algeriensis* aerial parts than all the other five tested strains (IZ = 9.33–17 mm) including *Salmonella* sp. and *S. aureus* CFSA2. Similarly, it was found that *Bacillus cereus* (ATCC10876), *Micrococcus luteus* (NRLL B-4375), and *Proteus mirabilis* (ATCC35659) were inhibited at 2.34, 7.03, and 4.68 mg/mL, respectively, of the EO extracted from the aerial parts of *T. algeriensis* growing in the north of Tunisia [68]. In addition, Zaïri et al. revealed that the best antibacterial activity was obtained using the EO from *T. algeriensis* leaves (MIC = 0.5 mg/mL) which also inhibited the fungus *Aspergillus flavus* by up to 42.86% [69]. Moreover, Rezzoug et al. [23] and Guesmi et al. [14] observed that the EO from the aerial parts of Algerian and Tunisian *T. algeriensis* demonstrated better antimicrobial activity that the ethanolic and aqueous extracts, respectively. For instance, *S. epidermidis* ATCC12228 and *S. aureus* ATCC25923 were inhibited at 128 *μ*g/mL using the methanolic extract and at only 32 *μ*g/mL using EO. More importantly, the antimicrobial efficiency of *T. algeriensis* EO from Libya was shown to be stronger than reference antibiotics such as ampicillin and streptomycin [6]. Additionally, Messaoudi et al. reported that the aerial part's ethanolic extract of *T. algeriensis* collected in the Algerian southwest has no effect on the growth of *E. cloacae* ATCC 49452 while it inhibited widely the growth of both *E. faecalis* ATCC 49452 and *S. aureus* ATCC 25923 [70]. However, aqueous extracts from leaves and aerial parts of Algerian *T. algeriensis* showed no inhibitory effect (no halo zone) against five human pathogens. Using methanol as the solvent, the leaves of *T. algeriensis* from the upper arid area of Tunisia showed a bacteriostatic effect against seven human pathogenic bacterial strains (MIC = 1.4 mg/mL) while the bactericidal activity was restricted to *E. coli* [71, 72].

Extract characterization revealed that samples containing carvacrol elicited a remarkable antibacterial activity [21]. Elsewhere, Nikolić et al. found a positive correlation between the antimicrobial activity of selected EO of *T. serpyllum*, *T. algeriensis*, and *T. vulgaris* and their chemical composition, which indicated that the activity may be ascribed to the presence of thymol because it occurs in high proportions [73].

The synergistic combination effect of *T. algeriensis* EO from Morocco and heat or pulsed electric fields against *E. coli* and *L. monocytogenes* was shown to inactivate the bacterial growth by of 5 log_10_ cycles [66]. Moreover, *T. algeriensis* EO were capable of inactivating 4–5 log_10_ cycles of the initial cell populations (*E*. *coli* O157:H7 VTEC (phage type 34) and *L*. *monocytogenes* (EGD-e)) in combination with high hydrostatic pressure [74]. Comparatively to other plants such as *E. globulus* and *R. officinalis*, EO from *T. algeriensis* were the most potent in inhibiting seven human pathogenic bacteria [75].

Plant pathogens such as *Phytophthora megakarya* (black pod of cocoa) were dose-dependently inhibited using both aqueous and ethanolic extracts of *T. algeriensis* leaves bought at a local market in Cameroon and completely inhibited at 60 and 125 mg/mL, respectively [76]. Similarly, *Botrytis cinerea* was inhibited by the EO from wild Tunisian *T. algeriensis* at >300 *μ*L/L [77]. Another investigation showed that 0.5% EO of *T. algeriensis* collected in northern Algeria decreased the rate of infestation of *Varroa destructor*, causing a mortality rate of 32.6% [78] (Table 6). Taken together, the EO of *T. algeriensis* are more potent than the different other extracts against human and plant pathogenic strains as well as other microbial species. Antibacterial activity of other Moroccan *Thymus* species was also reported in the literature such as *T. bleicherianus* [79], *T. serpyllum* [56], *T. munbyanus* [80], *T. hyemalis*, *T. vulgaris* [54], and *T. zygis* [81].

The mechanisms by which plant extracts trigger microbe survival and growth involve the integrity and the permeability of the membranes as well as the efflux pump systems of resistant Gram-negative bacteria such as *E. coli*, *E. aerogenes*, *K. pneumoniae*, *P. aeruginosa*, and *S. enterica Typhimurium* [82–84]. These effects are often explained by the hydrophobic nature of plant extracts allowing them to accumulate in bacterial cell membranes and disrupt their structure. In addition, the ability of some compounds to chelate transition metals such as copper and iron reduces their bioavailability to microorganisms [85]. Some researchers have observed that the antibacterial activity is correlated with the high contents of monoterpenes, phenols, aldehydes, and ketones in plant extracts that affect the cell membrane of microorganisms [86]. Others have been reported to induce a deficiency in microbial enzyme systems due to leakage of intracellular constituents leading to apoptosis [87]. Plant extracts can also trigger the phospholipids of the bacterial cell membrane, coagulate the cytoplasm, and attack lipids and proteins [88]. For instance, a study reported that *Salix tetrasperma* stem bark extract impaired *P. aeruginosa* virulence by inhibiting swimming and swarming mobilities as well as by reducing its hemolytic and proteolytic activities [89]. The antibacterial activities of plant extracts have been associated with several bioactive compounds such as polyphenols and molecules, namely, furocoumarins and furanocoumarins [85]. In general, the nature of a phytochemical may influence its mechanism of action and therefore its antibacterial activity. Finally, most antimicrobial activities of *T. algeriensis* extracts have been tested *in vitro*. However, it is interesting to evaluate their potential *in vivo* using animal models.

The antimicrobial effects of *T. algeriensis* extracts against human pathogenic bacteria and fungi make it an important plant to be explored using fractionation and testing procedures in order to isolate potentially active molecules that can be tested *in vivo* and through clinical trials.

### 6.4. Anticancer and Cytotoxic Activity

Nowadays, cancer is the most lethal disease [90]. Although several chemotherapeutic molecules are being used to manage cancer, development of drug resistance, problems of selective toxicity, and severe side effects are still challenging. Therefore, discovering new anticancer drugs is becoming more and more imperative [91]. In this regard, plant-derived compounds are a promising source of chemotherapeutic drugs. In the market today, many of the currently used chemotherapeutic medicines such as taxanes (docetaxel and paclitaxel), vinca alkaloids (vincristine, vinblastine, and semisynthetic drugs vindesine and vinorelbine), camptothecin derivatives (camptothecin and irinotecan), and epipodophyllotoxins (teniposide and etoposide) were initially isolated from medicinal plants [92].

The cytotoxic activity of *T. algeriensis* against cancer cells has been extensively studied using the methyl tetrazolium test (MTT), sulforhodamine B assay, and histopathological analysis (Table 4). Cytotoxicity was mostly assessed using EO. According to Ouakouak et al. [42], leaves' EO from the Algerian *T. algeriensis* showed high toxicity against the HCT116 tumor cell line (LC_90_ = 59.6 *μ*L/mL) while limited effect was seen towards the HePG2 hepatocellular carcinoma cell line (LC_90_ > 100 *μ*L/mL). Similarly, it was reported that the EO of *T. algeriensis* leaves and flowers exhibited cytotoxic activities at concentrations higher than 400 *μ*g/mL with CC_50_ = 725.92 ± 195.25 *μ*g/mL and 733.53 ± 141.96 *μ*g/mL, respectively [69]. Toxicities against other tumor cells such as NCI-H460 (non-small cell lung cancer), MCF-7 (breast), HCT-15 (colon), and AGS (gastric) were also reported using Libya's *T. algeriensis* EO with CC_50_ ranging from 62.12 to 64.79 *μ*g/mL. However, no toxicity was noticed on the porcine liver primary cell lines at concentrations > 400 g/mL [73]. Noteworthy, Jaafari et al. observed that the EO from the chemotype-rich Moroccan *T. algeriensis* which is carvacrol were more toxic than the other chemotypes' EO against the p815 mastocytoma tumor model cell line (up to 100% lysis) and had an important proliferative effect on the peripheral blood mononuclear cells (PBMC) [93]. Besides EO, other *T. algeriensis* extracts were also shown to be potent against cancer cell lines. For instance, the leaf methanolic extract of Algerian *T. algeriensis* was slightly toxic on A431 and SVT2 cancer cells at 100 *μ*g/mL. However, biocompatibility on both the immortalized tested cell lines HaCaT and BALB/c-3T3 was observed [47]. The anticancer potential of Tunisian *T. algeriensis* extracts of leaves and flowers on the HCT116 cell line revealed that both the aqueous and methanolic extracts are toxic with CC_50_ (*μ*g/mL) of 516.81 ± 47.42 and 528.05 ± 31.37, respectively. However, using *T. capitatus* and *R. officinalis* extracts, the aqueous extracts showed very low toxicities compared with the methanolic extracts [19].

The mechanisms underpinning the cytotoxic activity against cancer cells were linked to the nature of secondary metabolites in the plant extracts. For instance, berberine alkaloids are known to activate the apoptosis-inducing enzymes (caspase 3/9) of the human leukemia HL-60 cells by downregulating telomerase activity and nucleophosmin/B23 [94]. Additionally, some plant molecules such as curcumin have the potential to downregulate miR-21 expression in MCF-7 cells through the upregulation of the PTEN/Akt signaling pathway [95]. The anticancer potential of phytochemicals and their regulatory aspects were exhaustively described by Khan et al. [94]. In 2021, a study showed that even at low concentrations, *T. algeriensis* (from Tunisia) oily fraction inhibited HCT116 cell growth. Cleavage of poly(ADP-ribose) polymerase (PARP) and activation of the initiator and effector caspases show that *T. algeriensis* causes apoptotic cell death (caspases 3, 8, and 9). It also increased the expression of death receptors (DRs) while decreasing the expression of TNF-*α*-related apoptosis-inducing ligand (TRAIL) decoy receptors, according to the findings (DcRs). This study also showed that *T. algeriensis* increased MAPK pathway signaling molecules (p38 kinase, ERK, and JNK), downregulated c-FLIP, and overexpressed SP1 and CHOP. The *in vivo* model of cancer showed that intragastric injection of *T. algeriensis* extract (12.5 and 50 mg/mL) inhibited colorectal carcinogenesis in an animal model by preventing multiple phases in the carcinoma [96]. To sum up, the anticancer potential of *T. algeriensis* extracts is mainly attributed to their capacity to enhance the apoptosis-stimulating enzymes and receptors in tumor cells, to downregulate the oncogenic miRNA and genes, and to inhibit the antiapoptotic proteins. The anticancer and cytotoxic activity of *T. algeriensis* extracts is evident as has been widely proven. It turns out that this plant hides an enormous potential in the induction of apoptosis of cancer cells; that said, it will be extremely interesting to go further through *in vivo* and clinical experiments and to identify, like the compounds mentioned above, potential anticancer molecules with promising medical applications.

### 6.5. Miscellaneous Activities

*T. algeriensis* appears to be endowed with other biological activities such as antihemolytic, antiacetylcholinesterase, antilipase, insecticidal, leishmanicidal, and angiotensin converting enzyme (ACE) inhibitory activities (Table 4). Using erythrocyte osmotic fragility test, petroleum ether, chloroform, and n–BuOH extracts of the Algerian *T. algeriensis* (aerial part) induced resistance of erythrocytes to hemolysis. The highest antihemolytic effect was noted using the n–BuOH extract (IC_50_ = 322.85 ± 0.87 *μ*g/mL), followed by the chloroform extract (IC_50_ = 443.25 ± 0.52 *μ*g/mL). The petroleum ether extract displayed the lowest activity with 19.51% inhibition at 800 *μ*g/mL [44]. The antihemolytic effect of *T. algeriensis* extracts could be linked to their chemical composition, particularly flavonoids and polyphenols. In fact, previous research has shown that the presence of polyphenols and flavonoids in the crude extracts enhanced the stability of the erythrocyte membrane and inhibited hemolysis [97]. However, the exact mechanism of action of the membrane stabilization by the extract is yet to be elucidated [98]. Studies have also related the antihemolytic effect to the occurrence of molecules having antioxidant activities due to their ability to scavenge free radicals [99].

Previous studies showed that the methanolic extract from the *T. algeriensis* leaves (Tunisia) showed various abilities to inhibit acetylcholinesterase (AChE) with significant variations among *T. algeriensis* populations. It was observed that populations growing in the arid zone were the most potent (EC_50_ = 0.1–0.2 mg/mL) [21]. The modulation of the activity of AChE has been linked to the phenolic compounds [100, 101]. These latter effects have been reported to protect against neurodegenerative diseases [102]. For instance, rosmarinic acid was reported as an A*β* aggregation inhibitor [103, 104]. Besides, Benguechoua et al. showed, for the first time, that an acetone/water extraction mixture with ethyl acetate and butanol fractions from Algerian *T. algeriensis* inhibited *Candida rugosa* lipase [105].

Leaves' EO from Tunisian *T. algeriensis* used as a fumigant exhibited insecticidal activity against *Spodoptera littoralis* Boisd. with LC_50_ ranging from 41.75 to 131 *μ*L/L air [28]. The insecticidal effect of *T. algeriensis* EO is likely due to their major constituents such as *α-*pinene, 1,8-cineole, caryophyllene oxide, camphor, linalool, camphene, and *p-*eugenol, which were found to be toxic in many previous studies [106, 107]. This level of larvae mortality is higher than those observed using other medicinal plants such as *Thymus mastichina*, *Origanum majorana*, and *Salvia sclarea* [108]. According to Al-Nagar et al., terpene compounds constitute a major group of insect feeding deterrents and display a very strong inhibitory activity against larvae of *S. littoralis* [109]. Tunisian OE from the *T. algeriensis* aerial parts were also endowed with leishmanicidal activity against two Leishmania species: *L. infantum* and *L.* major with IC_50_ of 0.25 and 0.43 *μ*g/mL, respectively [49]. Some investigations indicated that the plant extracts including EO can target the metabolic pathways involved in Leishmania species survival such as MAP kinases, glyoxalase, metacaspases, and sterol pathways [110, 111]. These plant-based compounds include flavones, azoles, and staurosporine [111, 112]. Similarly, Melo et al. demonstrated that oleanolic acid, which possessed a good activity against *L. amazonensis*, *L. braziliensis*, and *L. infantum* (IC_50_<70 *μ*M in promastigotes and amastigotes), did not provoke FN-*γ*–assisted macrophage activation [113]. These findings indicated that this type of triterpene most likely acts on targets located in *Leishmania* parasites rather than in the host's macrophages.

Finally, Zouari et al. showed that *T. algeriensis* EO could be used as angiotensin I-converting enzyme (ACE) inhibitor for hypertension treatment (IC_50_ = 150 *μ*g/mL) [29]. To elucidate the mechanisms underlying ACE inhibition, it was suggested that the flavonoids such as vitexin and isovitexin isolated from various plants that inhibited ACE activity at 0.33 mg/mL by 20% and 45%, respectively, act by competing with the substrate for the ACE active site [114]. In addition, flavonoids are reported to form chelate complexes with the zinc atom within the active site of zinc-dependent metallopeptidases to which ACE belongs [115, 116].

It can be concluded that *T. algeriensis* extracts act as an antihemolytic by protecting the integrity of the erythrocyte's membrane, as antiacetylcholinesterase agents by inhibiting the A*β* aggregation, as an insecticidal and leishmanicidal by targeting the metabolic pathways for insects' survival, and as angiotensin converting enzyme (ACE) inhibitors by competing with the ACE active site. In the light of these various potentialities, some of which are being used traditionally, it would be original to investigate *T. algeriensis* extracts more deeply for these activities to spotlight the underlying mechanisms both *in vitro* and *in vivo*.

## 7. In Vivo Pharmacological Activities

In the biological systems, there is a growing need for *in vivo* experiments as most studies focus on *in vitro* and cell culture-based assays. However, these studies require verification using animal experiments to avoid misleading data and toxicity issues [117]. Indeed, the *in vivo* studies carried out on *T. algeriensis* extracts were comparatively limited and were only performed on species growing in Algeria and Tunisia using aerial part and leaf hydromethanolic extracts and EO. Reported studies are summarized in Table 7.

### 7.1. Safety Studies

As the interactions of plant constituents with biological components such as tissues, cells, proteins, and DNA could disrupt host's immune response and metabolism, assessing their *in vivo* behavior would provide a basis for beneficial as well as toxic responses and, more importantly, could lead to predictive models to assess their pharmacokinetics and intended effects [117].

Here, the *in vivo* toxicological evaluation of *T. algeriensis* extracts was investigated using animal models mainly *Albino* male mice and Sprague Dawley rats. Toxicity parameters were mostly related to hepatic and renal functions, mortality, body and organ weights, and histopathological evaluation of tissues (Table 7). Righi et al. showed that the hydromethanolic extract of *T. algeriensis* aerial parts (Algeria) administrated orally to *Albino* male mice at 200–800 mg/kg did not influence the hepatic (AST and ALT) and renal functional markers (urea and creatine) [24]. Similarly, Guesmi et al. observed no sign of toxicity or mortality after 15 days of oral administration of EO from Tunisian *T. algeriensis* at 180 mg/kg per day dissolved in normal saline to Sprague Dawley rats with no pathological change and normal histoarchitecture [118]. In another study, they showed that *T. algeriensis* EO exhibited no signs of acute toxicity with the absence of abnormal organic damage to the rats' organs and prevented H_2_O_2_-induced liver, kidney, and organ weight loss [20].

The leaf aqueous extract of Algerian *T. algeriensis* administered by oral route to Albino Wistar male and female mice at the doses of 2000 mg/kg and 5000 mg/kg induced no significant differences in body weight, no mortality, and/or no signs of toxicity. In addition, treated mice showed no change in the biochemical parameters and no histological abnormalities in their organs compared to control animals [119].

The major challenge encountering plant-derived agents is the prediction of their trajectory and location after *in vivo* administration. Hence, the relationship between the properties of a molecule and its distribution and behavior with the biological system should be studied as it is developed by pharmaceutical approaches for drugs. This is known as structure–function/toxicity relationship models [120]. Such data could be investigated before evaluating the systematic effect [117].

According to these studies, it seems that *T. algeriensis* is far from being toxic at least in tested models and at administered doses. However, more in-depth studies using pharmacokinetics and downstream targets of hepatic metabolites resulting from its compounds will have to be explored to ensure its biosafety for humans.

### 7.2. Antioxidant Activity

As mentioned above, *T. algeriensis* extracts are reported to be endowed with a high antioxidant activity *in vitro*. This was corroborated using *in vivo* experiments especially by Guesmi et al. [20, 118, 121, 122]. Various methods were used to investigate the antioxidant effect *in vivo*, mainly the assessment of the antioxidant machinery (enzymatic and nonenzymatic antioxidants), lipid peroxidation, and ferric reducing ability of plasma (Table 7). For instance, in 2016, these researchers demonstrated that Tunisian EO administrated orally to Sprague Dawley rats (150 mg/kg per day) subjected to low- and high-dose H_2_O_2_ treatment recovered the catalase (CAT), superoxide dismutase (SOD), and glutathione peroxidase (GPx) activities and glutathione (GSH) levels in liver and renal tissues of treated animals. In another study, they showed that *T. algeriensis* EO administered to adult male Wistar rats preserved the sperms' viability, improved the morphological abnormalities, and prevented induced DNA fragmentation in their testis. An increase in total protein in the H_2_O_2_-treated group was also noticed [121]. These researchers' investigations corroborated the same finding [20, 118]. Moreover, it was reported that the aerial part hydromethanolic extract of Algerian *T. algeriensis* increased the plasma antioxidant levels by 3-folds following treatment of Albino male mice with the dose of 800 mg/kg. At the same concentration, the iron reducing ability (908 *μ*M FeSO_4_eq/mL) was twofolds higher than that of the untreated group (405 *μ*M FeSO_4_/mL). In addition, a significant improvement in CAT activity and GSH levels was seen in mice treated with 400 and 800 mg/kg compared to the nontreated group accompanied with a significant decrease in the MDA levels in the plasma of treated groups by 200 and 400 mg/kg [24]. To sum up, *T. algeriensis* extracts displayed substantial antioxidant activities in several experimental models (*in vitro*: DPPH, FRAP, *β*–carotene bleaching, ORAC, TBARS, RP, phosphomolybdenum, lipid peroxidation inhibition, TAC, HRS, metal ions chelation, and superoxide anion scavenging assays, and *in vivo*: rats and mice) via diverse molecular targets/mechanisms by increasing the activities of antioxidant enzyme activities such as SOD, GST, and CAT and nonenzymatic antioxidants like GSH, reducing the MDA levels, and preventing the DNA damage. All these protective effects of *T. algeriensis* extracts against oxidative stress sustain the *in vitro* findings and highlight its benefits in the prevention and protection against oxidative stress and its radicals in related disorders and diseases.

### 7.3. Anti-Inflammatory Activity

The *in vivo* effectiveness of *T. algeriensis* as a source of anti-inflammatory agents has been investigated by Sobeh et al. using the leaf extracts of Algerian species [22] (Table 7). The study showed that the oral administration of the 80% MeOH extract to male rats injected with carrageenan induced a significant mild reduction in edema thickness using the doses of 200 and 400 mg/kg compared to the control rats. In addition, the increased vascular permeability induced by acetic acid injection in mice was reduced by 63 and 58% when animals were pretreated with *T. algeriensis* extracts at 400 or 600 mg/kg, respectively. These effects on the first phase of inflammation were attributed to the capacity of *T. algeriensis* extracts to inhibit the vasodilatation and the release of inflammatory mediators. In conclusion, *T. algeriensis* extracts exhibited prominent anti-inflammatory activities both *in vitro* and *in vivo* by inhibiting the inflammatory mediators such as COX, LOX, PGE2, LT, IL-1*β*, TNF-*α*, and IL-6 and by increasing the levels of Nrf–2 which controls the intracellular redox environment and redox homeostasis genes. The *in vivo* effects corroborate the anti-inflammatory activities reported *in vitro* and open promising prospects in using *T. algeriensis*-based compounds and derivatives in treating inflammatory related disorders in humans.

### 7.4. Leukocyte Recruitment

The effect of *T. algeriensis* leaf extracts on carrageenan-induced leukocyte migration into the peritoneal cavity in mice was also evaluated by Sobeh et al. [22] (Table 7). Following intraperitoneal injection of carrageenan, mice pretreated with *T. algeriensis* extract doses (200, 400, and 600 mg/kg) showed a dose-dependent decrease in the number of total leukocytes with up to 62%. This effect was more important that those observed using diclofenac and dexamethasone, which induced 39 and 30% decrease in leucocyte number, respectively. The observed reduction in leucocyte number has been explained by the suppression of inflammatory mediators such as prostaglandins and proinflammatory cytokines. It was also shown that the inhibition of leukocyte migration could be the result of the suppression of adhesion molecule and/or inhibition of the chemotactic of substances' expression [123]. This investigation confirms the *in vitro* anti-inflammatory activity of *T. algeriensis* extracts and highlights its positive effect on leukocyte migration and inflammation mediation during the immune response process.

### 7.5. Antinociceptive Activity

The central and peripheral analgesic activity of *T. algeriensis* leaf extract was studied in mice using the hot plate test and acetic acid-induced abdominal writhing assay, respectively [22] (Table 7). The study showed that oral pretreatment of mice with *T. algeriensis* extracts at 200 and 400 mg/kg prior to acetic acid injection induced a significant peripheral analgesic activity with a dose-dependent reduction in induced writhes. The effect observed by the dose 400 mg/kg (94% reduction) was higher than that achieved by dexamethasone (2 mg/kg) or diclofenac (20 mg/kg). Moreover, central antinociceptive activity was also shown using the extract's doses represented by a longer response latency. In addition, the extract at 400 mg/kg showed similar activity to the reference narcotic analgesic (nalbuphine). It was presumed that the leaf hydroalcoholic extract of *T. algeriensis* could act on the opioid system and/or other central nervous system (CNS) receptors involved in the antinociception. To sum up, *T. algeriensis* extracts exerted an antinociceptive effect *in vivo* by targeting the opioid system receptors (i.e., mu, delta, and kappa) and CNS nociceptors. This prominent analgesic effect of *T. algeriensis* extracts arouses a lot of interest in the search for metabolites endowed with antinociceptive activity which will certainly lead to good research and application perspectives of plant-based painkillers from *T. algeriensis*.

### 7.6. Gastroprotective and Antiulcerogenic Activity

The effect of *T. algeriensis* EO was investigated on Tunisian species using induced gastric lesions in adult male and female Wistar rats by 0.3 M HCl/60% ethanol [20] (Table 7). The results showed that oral treatment of rats by the EO (54–180 mg/kg, p.o.) induced a dose-dependent decrease in gastric lesions and reduced the ulcer index and percentage of inhibition mainly at doses of 180 mg/kg for male rats (88%) and between 117 and 180 mg/kg for female rats (96.25 and 98.85%). In addition, the gastric acidity of female rats pretreated with EO was significantly reduced comparatively to the ulcer control rats. The mucosal damage induced by HCl/ethanol in the control group was alleviated using the *T. algeriensis* EO. In fact, mucus production increased significantly in both male and female rats treated with 180 mg/kg. As compared to the acidified group in which visible change in the gross appearance of the gastric mucosa was seen, stomachs of male and female rats treated with the dose of 180 mg/kg showed a normal appearance. The authors suggested that the antiulceration effect of EO from *T. algeriensis* is attributed to their antioxidant effect and inhibition of neutrophil infiltration into ulcerated tissues. Nevertheless, the molecules and their structural aspects determining the observed effect are not yet established. The gastroprotective and antiulcerogenic activities of *T. algeriensis* EO seen in animal models explain its uses in traditional medicine for this purpose and open a great potential of using this plant's volatile constituents in the treatment of peptic ulcers and gastric troubles.

### 7.7. Neuroprotective Activity

The effects of *T. algeriensis* extracts on chronic neuropathic pain and the underpinning mechanisms were investigated by Rezq et al. [47] (Table 7). Using the chronic constriction injury (CCI) model in male Wistar rats, 80% MeOH leaf extracts of Algerian *T. algeriensis* (200 mg/kg and 400 mg/kg) prominently attenuated hyperalgesia and allodynia at day 14 of surgery. Noteworthy, the extracts showed a higher effect than the standard drug (pregabalin) towards both stimuli. In addition, they restored the normal appearance of the nerve fascicle which regained its regular form. Finally, the degeneration of most neurons caused by CCI was restored by the *T. algeriensis* extract at 400 mg/kg with few pyknotic neurons and low and deeply stained nuclei. The extracts also mitigated the increase of caspase 3 and synaptophysin. The underlying mechanisms of the observed neuroprotective activity were (i) inhibition of NOX–1, iNOS; (ii) increase of catalase activity; and (iii) inhibition of inflammatory mediators such as TNF–*α*, NF–*κ*B, COX–2, and PGE2. These effects suggest the substantial potential of *T. algeriensis* extracts in improving painful peripheral neuropathy. Furthermore, another earlier study on hydrophobic fractions of *T. algeriensis* (HFTS) growing in Tunisia showed that HFTS mitigates neuroinflammation via AChE inhibition and attenuates H_2_O_2_-induced brain toxicity [118]. In conclusion, *T. algeriensis* attenuated neuropathic pains and degeneration of neurons via antioxidant and anti-inflammatory properties. This opens new avenues for research of molecules exhibiting neuronal activities. Hence, bio-guided assays on *T. algeriensis* extracts can be envisaged with the aim of isolating molecules with neuroprotector power, unraveling their functional features, identifying their targets, and understanding their mode of actions.

### 7.8. Hot Topic Analysis of *T. algeriensis*

**Figure 7:**
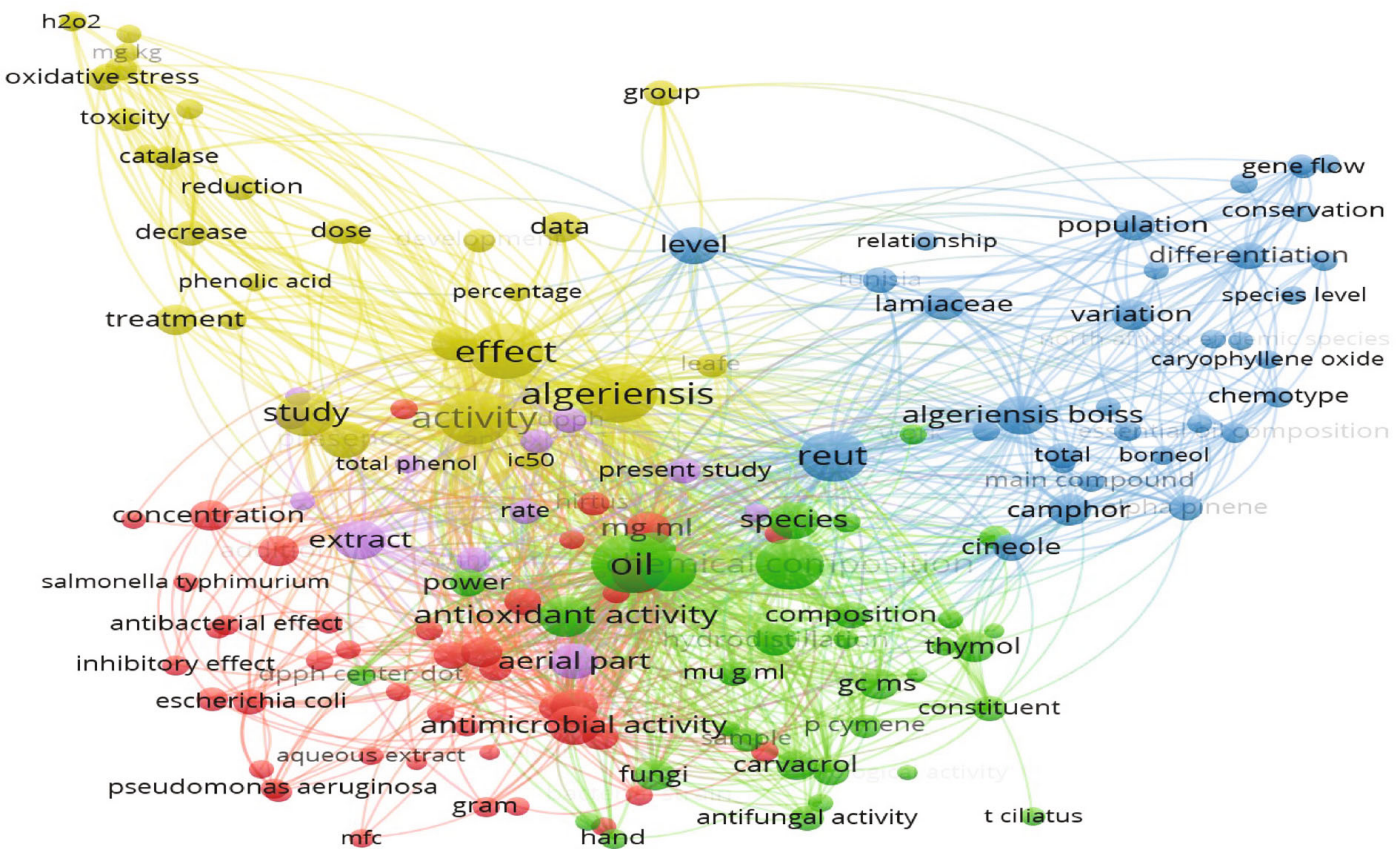
Coword map network visualization of *T. algeriensis*.

**Figure 8:**
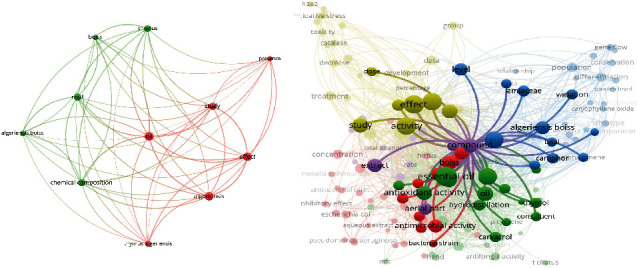
Knowledge map of *T. algeriensis*.

In this study, the bibliometric data used for the mapping tool is from the Web of Science database. The cooccurrence analysis of more than 50 high frequency keywords has been made to construct a knowledge map of the main strong domain of research related to *T. algeriensis*. In the visualization results, each keyword is represented by a circle. From the analysis of Figure 7, four topic groups are identified, which are “the different chemotypes of the plant,” “the chemical composition of the essential oil,” “the biological activities,” and the” *in vivo* activities.” Among them, the field of biological activities is the link between the field of chemical composition and the chemotype of the plant as there is a close relationship with these fields. After further domain structure detection as shown in Figure 8, the main topics in the field of biological activities of *T. algeriensis* were the antibacterial and antioxidant activities, directly related to the richness of the essential oil of the aerial parts of the plant in thymol, carvacrol, and *p*-cymene. The occurrences of these compounds, their absence, or their presence determine the chemotype of the plant.

## 8. Discussion

To the best of our knowledge, this is the first review to summarize and critically evaluate the chemical composition, biological and pharmacological activities, efficacy, and safety of *T. algeriensis* extracts and essential oils. *T. algeriensis* is one of the most well-known *Thymus* species in northern Africa. Most of the prior research on this plant species has focused on its biological activities and pharmacological and preclinical characteristics. Many chemical constituents have been identified from *T. algeriensis* extracts such as flavonoids, mainly kaempferol and rutin, besides luteolin, apigenin, hesperidin, and neohesperidin among others. They also contain phenolic acids such as rosmarinic acid and vanillic acid. Other constituents include sterols (e.g., *β*-sitosterol) and triterpenoid (e.g., oleanolic acid) [22, 23].

Current evidence revealed that the chemical composition of *T. algeriensis* essential oils (EO) differs according to its source. Its EO contains 40 volatile compounds represented mainly by monoterpenes and sesquiterpenoids. EO from *T. algeriensis* growing in Tunisia contains mainly 1,8-cineole and 4-terpineol [9, 15], while borneol, camphene, camphor, and carvacrol were listed from the Algerian and Moroccan plants. *p*-Cymene and thymol were detected in all flora. *β*-Myrcene (20.22%) has only been identified in the aerial parts of the Libyan plants [124], while myrcene characterized the EO of the Moroccan plants [53].

Free radicals are unstable and reactive because they have an unpaired electron. Superoxide, nitric oxide, and hydroxyl radicals, the most reactive and poisonous ROS, are among them. Hydrogen peroxide, singlet oxygen, and ozone are nonradical oxidants that produce free radicals in tissues through a variety of chemical processes. ROS are produced normally in mitochondria during aerobic respiration, by macrophages to fight infection, during induction of cytochrome p450, and by peroxisomes in the cells. Many exogenous factors can stimulate ROS production which damages cells and causes cellular injury and disease development such as tobacco smoke, ionizing radiation, UV light, industrial toxins such as carbon-tetrachloride, drugs, and charcoal-broiled foods [125]. Natural products with antioxidant properties such as *T. algeriensis* are beneficial in disease prevention and treatment. Antioxidant effects of *T. algeriensis* were investigated using different methods including DPPH, ABTS, FRAP, TBRS, TAC, HRS, and *β*-carotene bleaching assays [42, 126–128]. These studies showed that *T. algeriensis* extracts and EO had antioxidant properties. Furthermore, *in vivo* studies confirmed these *in vitro* tests. Although the *in vitro* studies investigated the antioxidant effects of this species, more comparative studies between extracts and EO of *T. algeriensis* obtained from various geographical areas are required. Moreover, *in vivo* antioxidant effects of *T*. *algeriensis* extracts and EO are not sufficient, and more studies are required.

**Figure 9:**
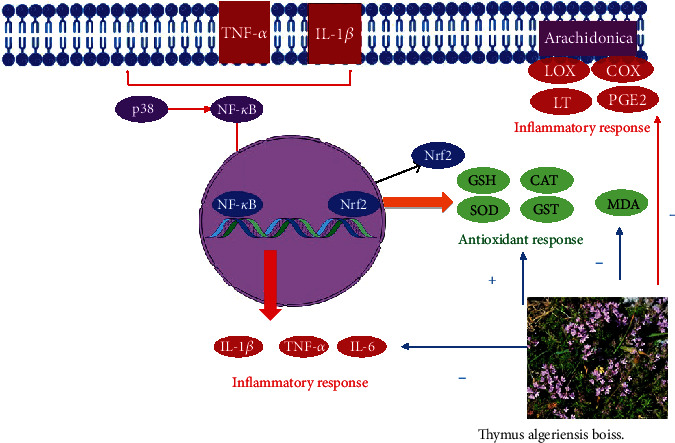
Mechanisms of antioxidant and anti-inflammatory effects of *T. algeriensis* extracts. Oxidative stress is associated with increased lipid peroxidation product (MDA) and decreased antioxidant enzyme activities such as SOD, GST, and CAT and nonenzymatic antioxidants such as GSH. Increased oxidative stress is a trigger for release of inflammatory cytokines such as TNF-*α* and IL-1*β* which increase p38 and subsequent phosphorylation and translocation of NF-*κ*B to the nucleus and subsequent transcription of genes responsible for formation of inflammatory cytokines creating a vicious cycle of inflammation. Furthermore, oxidative stress activates inflammatory enzymes such as LOX and COX with subsequent increase in both leukotriene and PG syntheses, respectively. *T. algeriensis* through its potent antioxidant effects can suppress all these inflammatory pathways and thus protect susceptible tissues against oxidative and inflammatory reactions. Abbreviations: LOX: lipoxygenase; COX: cyclooxygenase; LT: leukotrienes; PGE2: prostaglandin E2; GSH: reduced glutathione; GST: glutathione transferase; CAT: catalase; SOD: superoxide dismutase; MDA: malondialdehyde; IL-1*β*: interleukin-1*β*; IL-6: interleukin-6; NF-*κ*B: nuclear factor kappa B; Nrf–2: nuclear factor erythroid 2; p38: mitogen-activated protein kinase; TNF-*α*: tumor necrosis factor.

Inflammation is now recognized as a major factor in cellular and subcellular disorders. Few cells are engaged in this process, which boosts the production of proinflammatory chemical mediators (IL, TNF-*α*, NO, and PGs). Stimuli and tissue injuries influence mediator overproduction [129]. It was reported that phenolic compounds are similar to nonsteroidal anti-inflammatory drugs (NSAIDs) in their ability to decrease the chemical mediators of inflammation [130]. As mentioned previously in this review, *T. algeriensis* extracts and EO are rich in different phenolic compounds. *In vitro* studies showed their ability to inhibit inflammatory enzymes such as COX–1, COX–2, and 5-LOX [22, 44]. Besides, *in vivo* studies showed that the extracts suppressed the production of proinflammatory cytokines such as NF-*κ*B, TNF-*α*, lipoxygenase, COX–2 enzymes, and PGE2 [47, 131]. Additionally, the administration of *T. algeriensis* extracts to animal models enhanced their enzymatic and nonenzymatic antioxidant machinery (CAT, SOD, GPx, and GSH) in response to stress induction (Figure 9). Nevertheless, more studies on the different extracts and EO from *T. algeriensis* on inflammatory cell lines and *in vivo* models are still needed.

Moreover, *in vitro* studies showed that *T*. *algeriensis* extracts and EO had anticholinesterase activities [132], while *in vivo* studies on their use in treatment of Myasthenia gravis, Alzheimer diseases, or other diseases treated with anticholinesterases are lacking. In the same context, *in vitro* studies on ACE inhibition are promising [133], but no *in vivo* studies to our knowledge studied the antihypertensive effects or compared the extract effects with known ACE inhibitors (ACEIs). Similarly, *α*-glucosidase inhibitory effects of *T*. *algeriensis* extracts were explored, but no *in vivo* study on their antidiabetic effects was carried out [134].

Although gastroprotective, antinociceptive, anti-inflammatory, antioxidant, neuroprotective, and anticancer properties have all been documented for *T. algeriensis* extracts and EO, several studies are required on other disease models.

## 9. Conclusions and Future Perspectives

This review profiles the chemical composition of *T. algeriensis* and synthesizes the biological and pharmacological activities of its extracts and EO. Based on this comprehensive overview, the use of this plant's extracts in biotechnology seems to be promising because of their wealth in bioactive molecules mainly for antioxidant, antimicrobial, anticancer, anti-inflammatory, antinociceptive, gastro- and neuroprotective, and antiacetylcholinesterase purposes. However, as most of the biological activities have been evaluated *in vitro*, it would be interesting to extend their *in vivo* activities and uncover the underpinning mechanisms of action. In addition, the activities of *T. algeriensis* extracts vary depending on the solvents utilized. Moreover, environmental factors, geographical regions of growth, seasonal variations, phenological stages, degree of ripeness, plant part used, and harvesting time as well as postharvest treatment and processing could greatly influence the chemical composition of the plant and thus the related activities. This led to result inconsistency which represents a real issue for the development of bioactive preparations and their reliability. Thus, it could be interesting to study the effect of *T. algeriensis* domestication on its chemical composition and biological activities as it is a solution to conserve this plant species.

Since *T. algeriensis* was shown to improve sperm viability and mobility, further investigation could shed light on the potential estrogenic activity of this plant and its effect on reproductive hormones as Lamiaceae species including *Thymus* genus are traditionally used in Morocco to treat infertility disorders [135]. Additionally, as the revolution for patient safety has gained momentum importance, the safety of *T. algeriensis* cannot be definitively established from reported studies. More attention should still be given to the toxicity aspect in a more in-depth manner especially when using EO that could be harmful in some individuals. Although the preclinical investigations (*in vitro*, cell-based, and animal studies) on this plant species are available in a large quantity, they should be translated into evidence-based clinical progress using human trials which are completely missing. Having said that, there are many opportunities for the healthcare industry to explore the benefits of *T. algeriensis* because of the potential growth market of medicinal plants. Therefore, future prospective studies should screen fractions or individual constituents that exhibit health-protecting and curative activities from *T. algeriensis*.

## Figures and Tables

**Table 1:** Chemical constituents of *T. algeriensis* extracts.

Compound name	Extract type	Ref.
*Algeria*		
Apigenin-6,8-*C*-dihexoside^b^	H_2_O & EtOH–H_2_O	[7]
Apigenin-7-*O-*glucuronide^b^	H_2_O & EtOH–H_2_O	[7]
Aringin^a^	EtOH	[23]
Baicalin^b^	n–BuOH	[17]
Benzoic acid^b^	H_2_O	[18]
Caffeoyl rosmarinic acid^a^	MeOH–H_2_O	[24]
Chlorogenic acid^b^	H_2_O	[18]
Clovane-2,9-diol^a^	MeOH	[25]
Diosmin^b^	n–BuOH	[17]
Ellagic acid^a^	EtOH	[23]
Fumaric acid^b^	n–BuOH	[17]
Gentisic acid^b^	n–BuOH	[17]
Hesperidin^b^	n–BuOH	[17]
Isovanillin^b^	EtOH–H_2_O & H_2_O	[18]
Kaempferol-*O*-glucuronide^b^	H_2_O & EtOH–H_2_O	[7]
Lithospermic acid^b^	H_2_O & EtOH–H_2_O	[7]
Luteolin glucuronide^a^	MeOH	[22]
Methyl ursolate^a^	MeOH	[25]
Naringin^b^	EtOH–H_2_O, H_2_O, & n–BuOH	[17, 18]
Neohesperidin^b^	n–BuOH	[17]
*O*-Coumaric acid^b^	H_2_O	[18]
Oleanolic acid^a^	MeOH	[25]
*p*-Coumaric acid^b^	EtOH–H_2_O, H_2_O	[18]
Epicatechin^a^	EtOH	[23]
Rosmarinic acid glucoside^a^	MeOH	[22]
Salvianolic acid K^ab^	MeOH, H_2_O, & EtOH–H_2_O	[7, 22]
Scutellarin^b^	n–BuOH	[17]
Sinapinic acid^b^	EtOH–H_2_O, H_2_O	[18]
*t*-Ferulic acid^b^	H_2_O	[18]
Ursolic acid^a^	MeOH	[25]
*β*-Sitosterol^a^	MeOH	[25]
2,5-Dihydroxybenzoic acid^a^	EtOH	[23]
2,3-Dimethoxybenzoic acid^b^	EtOH–H_2_O, H_2_O	[18]
3-Hydroxybenzoic acid^b^	EtOH–H_2_O, H_2_O	[18]
4-Hydroxybenzoic acid^b^	H_2_O, EtOH–H_2_O, & n–BuOH	[18]
*Algeria and Tunisia*		
Catechin^b^	H_2_O	[14, 18]
Gallic acid^b^	H_2_O & MeOH	[18–20]
Kaempferol^ab^	MeOH–H_2_O, H_2_O, & MeOH	[14, 19, 24]
Quercetin^b^	H_2_O & MeOH	[14, 18, 19, 22]
Rosmarinic acid^ab^	MeOH–H_2_O & MeOH	[7, 21, 24]
Rutin^ab^	EtOH–H_2_O, H_2_O, & MeOH	[14, 18–20, 23]
Syringic acid^b^	H_2_O & MeOH	[18, 20]
Vanillic acid^ab^	EtOH, MeOH, & H_2_O	[18–20, 23]
Epicatechin^b^	EtOH–H_2_O & H_2_O	[14, 18, 19]
*Tunisia*		
Gallic acid^b^	H_2_O	[14]
Naringenin^b^		[14, 19]
Coumaric acid^b^		[14, 19]
Caffeic acid^b^	MeOH	[20]
Ferulic acid^b^		[20]
Flavone^b^		[20]
Hydroxyphenylic acid^b^		[20]
Methyl gallate^b^		[20]
(+)-Catechin hydrate^b^		[20]
Carvacrol^a^		[21]
Kaempferol-*O*-hexoside^a^		[21]
Kaempferol-*O*-hexuronide^a^		[21]
Apigenin^b^	H_2_O, MeOH	[14, 19]

^a^Leaves, ^b^aerial parts.

**Table 2:** Chemical constituents of *T. algeriensis* essential oils (EO).

Compound name	Plant part	Quantity (%)	Country	Ref.
1,8-Cineole	Aerial parts	17.70%	Tunisia	[9]
		7.55-22.07% ∗		[15]
		20.98%		[12]
		19.96%		[20]
	Leaves	11.60%		[28]
		12.05%		[69]
	Flowers	9.12%		[69]
	Leaves and flowers	5.54%		[29]
		5.16-11.21%^∗∗^	Algeria	[42]
		7.69%		[34]
		6.00%		[136]
		5.94%		[30]
4-Terpineol	Aerial parts	1.55-11.86%^∗^	Tunisia	[15]
	Leaves and flowers	7.36%		[29]
Borneol	Aerial parts	11.16-22.2%^∗∗^	Algeria	[42]
		5.74%		[136]
	Stem bark	11.16%		[30]
	Aerial parts	28%	Morocco	[137]
		18.30%		[53]
		23.48%		[66]
		59%		[93]
Camphene	Aerial parts	7.53-12.86%^∗∗^	Algeria	[42]
	Stem bark	12.78%		[30]
	Aerial parts	20.90%	Morocco	[137]
		11.80%		[53]
Camphor	Aerial parts	17.45-32.56%^∗∗^	Algeria	[42]
		13.62%		[136]
		14.22%		[46]
		17.68%		[67]
	Stem bark	22.60%		[30]
	Aerial parts	15.70%	Morocco	[137]
		10.00%		[53]
		27.70%		[50]
		27.70%		[79]
		27.70%		[26]
		19.20%	Tunisia	[20]
		6.8-19.93%^∗^		[15]
		7.46%		[12]
		13.82%		[49]
		8.20%		[9]
	Leaves	10.40%		[28]
	Leaves and flowers	7.82%		[29]
Carvacrol	Aerial parts	48.40%	Algeria	[78]
		28.10%		[138]
	Leaves	64.6-65.9%^¥^		[126]
		4%		[139]
	Aerial parts	80.90%	Libya	[6]
		14%		[73]
		4.59%		[43]
		36.78%		[124]
	Aerial parts	85%	Morocco	[93]
Caryophyllene oxide	Stems	17.80%	Tunisia	[28]
	Roots	21.10%		[28]
cis-Sabinene hydrate	Aerial parts	0.10-12.95%^∗^	Tunisia	[15]
	Leaves and flowers	5.29%		[29]
Elemol	Aerial parts	18.38%	Algeria	[46]
	Leaves	3.98%	Tunisia	[69]
	Flowers	11.30%		[69]
	Stems	10.20%		[28]
Geraniol	Aerial parts	19.60%	Algeria	[35]
	Leaves	7.30%	Morocco	[140]
Linalool	Aerial parts	3.93%	Algeria	[136]
		30.40%		[35]
		47.30%		[141]
		78.80%		[142]
		22.15%	Tunisia	[12]
		17.62%		[49]
	Leaves	3.20%		[69]
*p*-Cymene	Aerial parts	8.00%	Algeria	[138]
		20.04%		[67]
		14.70%		[78]
		6.80%		[141]
	Leaves	6.2-6.9%^¥^		[126]
		3%		[139]
	Aerial parts	7.70%	Libya	[6]
		8.91%		[43]
		23%	Morocco	[93]
		27.18%	Tunisia	[77]
Thymol	Aerial parts	20.83%	Algeria	[67]
		5.60%		[78]
		20.20%		[35]
		29.20%		[141]
		62.70%		[124]
	Leaves	71%		[139]
	Aerial parts	56.00%	Libya	[73]
		38.50%		[43]
		12.45%		[124]
	Aerial parts	42%	Morocco	[93]
	Aerial parts	36.94%	Tunisia	[77]
Viridiflorol	Aerial parts	4.00%	Algeria	[136]
		0-11.49%^∗^	Tunisia	[15]
	Roots	17.20%		[28]
*α*-Pinene	Aerial parts	6.80%	Algeria	[23]
		27.14%		[34]
	Stem bark	5.01%		[30]
	Aerial parts	20.50%	Morocco	[50]
		20.50%		[79]
		20.50%		[26]
		7.41-13.94%^∗^	Tunisia	[15]
		21.31%		[12]
		11.49%		[12]
		15.50%		[9]
	Leaves	19.50%		[28]
		2.97%		[69]
	Leaves and flowers	6.75%		[29]
*α*-Terpinene	Aerial parts	10.66%	Libya	[124]
		3.24%	Tunisia	[77]
		6.41%		[49]
*β*-Caryophyllene	Aerial parts	11.00%	Algeria	[143]
	Leaves	3.0-3.4%^¥^		[126]
*γ*-Terpinene	Aerial parts	14.90%	Algeria	[78]
	Leaves	5.9-6.7%^¥^		[126]
		0.50%		[139]
	Aerial parts	7.19%	Libya	[43]
		9.90%	Tunisia	[77]
*δ*-Cadinene	Aerial parts	4.00%	Algeria	[143]
		3.39%		[34]
*α* -Caryophyllene	Aerial parts	9.68%	Algeria	[46]
*α*-Terpinyl acetate		47.40%		[23]
*β*-Eudesmol		11.50%		[46]
Bornyl acetate		3.86-7.92%^∗^		[140]
*γ*-Terpinene		25.70%		[67]
Germacrene D		29.60%		[143]
Neryl acetate		9.60%		[23]
Eucalyptol		10.04%		[67]
Bicyclogermacrene		4.40%		[143]
-*β*-Farnesene		7.80%		[143]
2,3-Dehydro-1,4-cineol		36%		[144]
Linalyl acetate		6.39%	Tunisia	[12]
*α*-Humulene		5.72%		[12]
*α*-Terpenyl acetate		6.27%		[49]
*β*-Linalool		3.15%		[77]
Methyl eugenol		6.78%		[12]
Terpinen-4-ol		6.80%		[49]
Terpenyl acetate		0-14.92%^∗∗^		[15]
*β*-Myrcene		20.22%	Libya	[124]
Myrcene		8.60%	Morocco	[53]
trans-Caryophyllene	Leaves	2.40%	Morocco	[140]
Geranyl acetate		80.80%		[140]
Acorenone	Stem bark	5.84%	Algeria	[30]

^∗^Collected during the vegetative and flowering stages and from eight different geographic regions; ^∗∗^collected before, during, and after flowering stage; ^¥^effect of different gamma irradiation doses.

**Table 3:** *In vitro* antioxidant activities of *T. algeriensis* extracts.

Extract	Used method	Effects	Ref.
*Algeria*			
Aerial parts			
PE, CHCl_3_, & n–BuOH	DPPH	IC_50_ (mg/mL) = 69.50 ± 0.68 (PE), 79.92 ± 0.30 (CHCl_3_), and 5.05 ± 0.12 (n–BuOH)	[44]
	CUPRAC	A_0_._50_ (*μ*g/mL) = 22.28 ± 0.24 (PE), 27.81 ± 3.06 (CHCl_3_), and 0.94 ± 0.06 (n–BuOH)	
	RP	A_0_._50_ (*μ*g/mL) = 25.25 ± 0.08 (PE), 24.5 ± 0.52 (CHCl_3_), and 4.98 ± 0.48 (n–BuOH)	
	TAC	TAC (*μ*g EAA/mg (dw)) = 15.69 ± 0.001 (PE), 16.21 ± 0.02 (CHCl_3_), and 20.79 ± 0.19 (n–BuOH)	
	FTC	%of inhibition = 27.80 ± 0.37 (PE), 24.25 ± 0.45 (CHCl_3_), and 47.43 ± 0.58 (n–BuOH)	
EtOH & H_2_O	DPPH	IC_50_ (mg/mL) = 0.052 ± 0.004 (EtOH), not active (H_2_O)	[145]
	ABTS	IC_50_ (*μ*g/mL) = 42 ± 0.99 (EtOH), 52 ± 31 (H_2_O)	
MeOH–H_2_O	DPPH	IC_50_ (*μ*g/mL) = 7.4 ± 0.3	[24]
	Iron chelating	EC_50_ (*μ*g/mL) = 512 ± 0	
	*β*–Carotene bleaching	%of inhibition = 90 ± 2	
	TAC	TAC (*μ*g AAE/mg) = 268 ± 4	
	FRAP	FRAP (mM FeSO_4_/mg) = 5.3 ± 0.0	
H_2_O & EtOH–H_2_O	DPPH	EC_50_ (*μ*g/mL) = 64.8 ± 0.7 (H_2_O), 131 ± 3 (EtOH–H_2_O)	[7]
	RP	EC_50_ (*μ*g/mL) = 54.0 ± 0.5 (H_2_O), 100.2 ± 0.5 (EtOH–H_2_O)	
	*β*–Carotene bleaching	EC_50_ (*μ*g/mL) = 149 ± 3 (H_2_O), 85 ± 3 (EtOH–H_2_O)	
	TBARS	EC_50_ (*μ*g/mL) = 26.3 ± 0.2 (H_2_O), 40.3 ± 0.3 (EtOH–H_2_O)	
EA & n–BuOH	DPPH	EC_50_ (mg/mL) = 0.290 (EA) EC_50_ (mg/mL) = 1.45 (n–BuOH)	[46]
EO	DPPH	IC_50_ (mg/mL) = 10.2 ± 0.9–>45.0	[67]
	Phosphomolybdenum assay	AEAC (mg/mL) = 0.148 ± 0.003–0.220 ± 0.022	
MeOH–H_2_O, acetone–H_2_O, MeOH, acetone–H_2_O	DPPH, ABTS, phosphomolybdenum	All extracts possess potential antioxidant activities compared to standards	[105]
EO	TBARS	Not active	[62]
	ABTS	IC_50_ (mg/mL) = 0.150 ± 0.002	
	DPPH	IC_50_ (mg/mL) = 0.235 ± 0.018	
	ORAC	ORAC (*μ*mol Trolox equivalent/g) = 38.47 ± 39.71	
	RP	IC_50_ (mg/mL) = 0.025 ± 0.006	
	Chelating metal ions	Not active	
	HRS	Not active	
	Superoxide anion scavenging assay (nonenzymatic method)	Not active	
EO	HRS	IC_50_ (*μ*g/mL) = 2.2 ± 0.03–8.5 ± 0.1 (ALG1–ALG3)	[35]
	DPPH	%of inhibition = 1.6 ± 0.0–53.4 ± 0.2 (chemotype and dose-dependent effect)	
	TBARS	IC_50_ (*μ*g/mL) = 106.7 ± 8.4–911.6 ± 7.4 (ALG1–ALG3)	
Leaves			
MeOH	DPPH	EC_50_ (*μ*g/mL) = 1.60 ± 0.13	[45]
	*β*–Carotene bleaching test	%of inhibition = 64.31 ± 1.9	
Lipophilic extract using olive oil (OO)	RP_lip_	Significantly higher (RP_lip_ = 50 mg BHT eq/g (dw)) than that of OO only (40 mg BHT eq/g OO)	[146]
EtOH & EO	DPPH	IC_50_ (mg/mL) = 1.560 ± 0.010 (EtOH), 1.437 ± 4.51 (EO)	[23]
	ABTS	IC_50_ (mg/mL) = 1.743 ± 0.195 (EtOH), 0.8960 ± 0.203 (EO)	
	RP	AEAC–FRAP assay (*μ*g/mL) = 0.897 ± 0.064 (EtOH), 1.387 ± 0.265 (EO)	
	Phosphomolybdenum	AEAC (mg/mL) = 0.007 ± 0.0006 (EtOH), 0.432 ± 0.001 (EO)	
MeOH	DCFDA	No significant modification in ROS levels in HaCaT cells	[47]
	Western blot analyses	Significant increase in nuclear levels of Nrf–2 (nuclear factor erythroid 2) by up to 180% after incubation of the HaCaT cells for 15 min	
H_2_O & EO	DPPH	IC_50_ (mg/L) = 404.08 ± 5.87 (EO), 22.26 ± 0.07 (H_2_O)	[126]
	ABTS	IC_50_ (mg/L) = 10.48 ± 0.49 (EO), 25.29 ± 0.21 (H_2_O)	
	TBARS	IC_50_ (mg/L) = 23.54 ± 0.37 (EO), not active (H_2_O)	
	RP	IC_50_ (mg/L) = 347.84 ± 3.02 (EO), 59.53 ± 0.70 (H_2_O)	
EO	DPPH	IC_50_ (mg/mL) = 41.09	[42]
	ABTS	IC_50_ (mg/mL) = 10.84	
	TAC	TAC (U/L) = 39.27 ± 3.47	
Stem bark			
EO	DPPH	IC_50_ (mg/mL) = 83.8	[30]
*Tunisia*			
Aerial parts			
EO	DPPH	IC_50_ (mg/mL) = 0.8	[29]
	*β*–Carotene bleaching	IC_50_ (mg/mL) = 0.5	
EO	DDPH	IC_50_ (*μ*g/mL) = 3155 ± 27.56	[49]
EO aqueous extract	DPPH FRAP	IC_50_ (*μ*g/mL) = 0.06	[127]
		IC_50_ (*μ*g/mL) = 0.04	
H_2_O & hexane	DPPH	IC_50_ (*μ*g/mL) = 6.7 (H_2_O)	[14]
	FRAP	Samples at vegetative and flowering stages (200, 300, 400, and 500 *μ*g/mL) reduced the Fe^3+^ to Fe^2+^ with lower potency than BHT	
EO	DPPH	%of inhibition = 52–91.96%	[118]
EtOH & H_2_O	DPPH	IC_50_ (*μ*g/mL) = 43.5 ± 1.36	[147]
	FRAP	IC_50_ (*μ*g/mL) = 378.5 ± 5.24	
	*β*–Carotene bleaching	IC_50_ (*μ*g/mL) = 1430 ± 10.79	
EO & MeOH	DPPH	%of inhibition = 81 ± 0.26–93 ± 0.06 (MeOH), 82 ± 0.52–85 ± 0.57 (EO)	[20]
	ABTS	%of inhibition = 22 ± 0.9–75 ± 0.72 (MeOH), 8 ± 0.7–19 ± 0.33 (EO)	
	*β*–Carotene bleaching	%of inhibition = 25 ± 0.08–50 ± 0.12 (MeOH), 4 ± 0.44–10 ± 0.52 (EO)	
Leaves			
MeOH	DPPH	IC_50_ (*μ*g/mL) = 8.9 ± 0.1–68.8 ± 1.0	[21]
	FRAP	EC_50_ (mmol Fe^2+^/L) = 1.0 ± 0.0–20.6 ± 0.2	
	*β*–Carotene bleaching	EC_50_ (*μ*g/mL) = 0.03 ± 0.0–1.81 ± 0.0	
EO	DPPH	IC_50_ (mg/mL) = 4.31 ± 0.7–9.23 ± 1.8	[28]
	ABTS	ABTS (*μ*g Trolox equivalent/mg DW) = 11.69 ± 0.64–18.13 ± 0.92	
Leaves & flowers			
EO	DPPH	IC_50_ (*μ*g/mL) = 0.347 ± 0 (L), 0.349 ± 0 (F)	[69]
MeOH & H_2_O		IC_50_ (*μ*g/mL) = 3.46 ± 0.010–3.88 ± 0.015 (H_2_O), 3.13 ± 0.011–4.27 ± 0.010 (MeOH)	[19]
*Morocco*			
Aerial parts			
EA & MeOH	DPPH	IC_50_ (*μ*g/mL) = 14.8	[148]
	*β*–Carotene bleaching	IC_50_ (*μ*g/mL) = 59.85 ± 1.98	
EO	DPPH	IC_50_ (*μ*g/mL) = 745.6	[50]
		IC_50_ (*μ*g/mL) = 1800	[53]
EO	DPPH	IC_50_ (*μ*g/mL) = 67.85	[131]
		
Leaves			
H_2_O	DPPH	IC_50_ (*μ*g/mL) = 32.40	[140]
EO	DPPH	IC_50_ (*μ*g/mL) = 6.88	[149]
	ABTS	IC_50_ (*μ*g/mL) = 6.96	
*Libya*			
Aerial parts			
EO	DPPH	EC_50_ (mg/mL) = 1.64 ± 0.05	[73]
	RP	EC_50_ (mg/mL) = 0.68 ± 0.01	
	*β*–Carotene bleaching	EC_50_ (mg/mL) = 1.56 ± 0.12	
	TBARS	EC_50_ (mg/mL) = 0.31 ± 0.01	
	DPPH	EC_50_ (mg/mL) = 0.299	[43]
	DPPH	EC_50_ (mg/mL) = 0.132	[6]

ABTS: 2,2′-azino-bis 3-ethylbenzothiazoline-6-sulfonic acid; AEAC: ascorbic acid equivalent antioxidant capacity; BHT: butyl-hydroxytoluene; CUPRAC: cupric reducing antioxidant capacity; DCFDA: dichlorodihydrofluorescein diacetate; DPPH: 2,2-diphenyl-1-picryl-hydrazyl-hydrate; EA: ethyl acetate; EAA: equivalents of ascorbic acid; FRAP: ferric-reducing antioxidant power; FTC: ferric thiocyanate; HRS: hydroxyl radical scavenging; ORAC: oxygen radical absorbance capacity; PE: petroleum ether; RP: reducing power; TAC: total antioxidant capacity; TBARS: thiobarbituric acid reactive substances.

**Table 4:** *In vitro* activities of *T. algeriensis* extracts.

Extract	Activity	Used method	Country	Effects	**Ref**
*Aerial part*					
PE, CHCl_3_, and n–BuOH	Antihemolytic	Erythrocyte osmotic fragility	Algeria	IC_50_ (*μ*g/mL) = 19.51 ± 0.17 (PE), 443.25 ± 0.52 (CHCl_3_), 322.85 ± 0.87 (n–BuOH)	[44]
	Anti-inflammatory	Egg albumin denaturation		%inhibition = 30.26 (PE), 45.27 (CHCl_3_), 26.03 (n–BuOH)	
EA & MeOH	Anticorrosive	Gravimetric and electrochemical	Morocco	%inhibition = 87% (MeOH) IC_50_ (*μ*g/mL) = 59.85 ± 1.98	[148]
EO	Antitumor and cytotoxic	Sulforhodamine B	Libya	GI_50_ (*μ*g/mL) = 62.12–64.79	[73]
		Hepatotoxicity evaluation		None of the EO showed toxicity at tested concentrations (>400 g/mL) for porcine liver primary cell culture	
EO	Anti-inflammatory	5–Lipoxygenase	Algeria	IC_50_ (*μ*g/mL) = 0.083 ± 0.005	[62]
EO	Leishmanicidal	MTT assay	Tunisia	*L*.*infantum* IC_50_ (*μ*g/mL) = 0.25	[49]
				*L*.*major* IC_50_ (*μ*g/mL) = 0.43	
	Cytotoxic			IC_80_ (*μ*g/mL) = 0.67 (murine macrophages RAW264.7)	
EO	ACE inhibition	Spectrophotometry	Tunisia	IC_50_ (*μ*g/mL) = 150	[29]
EO	Anticorrosive	Weight loss measurement	Morocco	Inhibited the corrosion rate (*C*_R_) of mild steel at all concentrations (*C*_R_ = 0.012 (EO) versus 1.23 mg/cm^2^ h (blank) at 313 K)	[137]
		Potentiodynamic polarization		Acted as mixed-type inhibitor	
		Electrochemical impedance spectroscopy		Inhibition efficiency (*E*_RT_ (%)) = 46–93	
*Leaves*					
EO	Phytotoxic	*In vitro* seed germination inhibition (allelopathic effect)	Tunisia	100% inhibition of *M. sativa* at 1 mg/mL (dose-dependent effect)	[28]
	Insecticidal	Fumigant bioassay against *Spodoptera littoralis* Boisd.		LC_50_ (*μ*L/L air) (LLD–ULD) = 41.75–131 LC_90_ (*μ*L/L air) (LLD–ULD) = 53.25–189.25	
EO	Cytotoxic	Mitochondrial-dependent reduction of yellow	Algeria	LC_50_ (*μ*g/mL) = 39.8, LC_90_ (*μ*g/mL) = 59.6 (cytotoxic on HCT116 cell line)	[42]
				LC_50_ and LC_90_ (*μ*g/mL) > 100 (limited activity against the HePG2 cell line)	
MeOH	COX inhibition	EIA	Algeria	IC_50_ (*μ*M) = 12.4 ± 0.49 (COX–1), 0.05 ± 0.01 (COX–2)	[22]
	LOX inhibition	Lipoxygenase inhibitor screening		IC_50_ (*μ*M) = 2.70 ± 0.23	
MeOH	Cytotoxic	MTT	Algeria	Biocompatible on both the immortalized tested cell lines HaCaT and BALB/c-3T3 and slightly toxic on A431 and SVT2 cancer cells at high concentrations (100 *μ*g/mL)	[47]
MeOH	Silver nanoparticle biosynthesis	Dropwise addition of the plant extract to the silver nitrate solution	Algeria	The extract acts as a reducing as well as a stabilizing agent Extract at 10% induced a consistent increase in the intensity of the surface plasmon peak absorbance for AgNPs	[71]
EtOH and EO	Anticancer	MTT	Algeria	LC_50_ (*μ*g/mL) ≥ 10,000 (EtOH), 300 ± 13–1067 ± 96 (EO)	[23]
MeOH	Acetylcholinesterase inhibition	Spectrophotometry	Tunisia	%inhibition = 94.5%	[21]
*Flowers & leaves*					
MeOH & H_2_O	Cytotoxic	MTT	Tunisia	CC_50_ (*μ*g/mL) = 508 ± 45.32–516.81 ± 47.42 (H_2_O), 520.12 ± 32.56–528.05 ± 31.37 (MeOH)	[19]
EO	Cytotoxic	MTT	Tunisia	CC_50_ (*μ*g/mL) = 725.92 ± 195.25 (L), 733.53 ± 141.96 (F)	[69]
EO	Anticancer	MTT	Morocco	Ta1 is more cytotoxic (100% lysis) than Tb2 (60% lysis) against P815 tumor cell line	[93]
	PBMC			Increased viability by 200%	

ACE: angiotensin I-converting enzyme; CC: half maximal cytotoxic concentration; COX: cyclooxygenase; EIA: enzyme immunoassay; IC_50_: half maximal inhibitory concentration; IC_80_: concentration resulting in 80% inhibition; LC_50_: half maximal lethal concentration; LC_90_: concentration resulting in 90% lethality; LOX: lipoxygenase; MTT: methyl tetrazolium test; PBMC: peripheral blood mononuclear cells.

**Table 5:** Antibacterial activities of *T. algeriensis* extracts.

Extract	Tested strains	Key results	Ref.
*Aerial part*			
MeOH–H_2_O	*S. aureus* ATCC 29213	Resistant to all the extracts	[44]
	*E. faecalis* ATCC 29212	MIC (*μ*g/mL) = 6.25 (n–BuOH) MIC (*μ*g/mL) = 12.5 (PE & CHCl_3_)	
	*E. coli* ATCC 25922	MIC (*μ*g/mL) = 25 (PE, CHCl_3_ & n–BuOH)	
	*P. aeruginosa* DMS 1117	Resistant to all the extracts	
EO	*E. coli* SB3	MBC (mg/mL) = 25, MIC (mg/mL) = 12.5	[150]
	*K. pneumoniae* SB4	MBC (mg/mL) = 25, MIC (mg/mL) = 12.5	
	*K. pneumoniae* SB5	MBC (mg/mL) = 25, MIC (mg/mL) = 3.12	
	*K. pneumoniae* SB6	MBC (mg/mL) = 25, MIC (mg/mL) = 1.56	
EO	*M. luteus* ATCC 9314	IZ (mm) = 18.0 ± 0.6	[42]
	*S. aureus* ATCC 43,300	IZ (mm) = 18.0 ± 0.7	
	*E. coli*	IZ (mm) = 13.0 ± 0.9	
EO	*S. aureus*	MIC (*μ*g/mL) ≤ 0.5	[75]
	*L. monocytogenes* (EGD-e)	MIC (*μ*g/mL) ≤ 0.5	
	*L. monocytogenes* (4b)	MIC (*μ*g/mL) ≤ 0.5	
	*E. faecalis*	MIC (*μ*g/mL) ≤ 0.5	
	*S. Enteritidis*	MIC (*μ*g/mL) = 1.0	
	*E. coli* O157:H7	MIC (*μ*g/mL) = 1.0	
	*P. aeruginosa*	MIC (*μ*g/mL) = 1.0	
EO	*L. monocytogenes* (ATCC 19118)	MIC (%) = 0.025, MBC (%) = 0.05	[68]
	*S. aureus* (ATCC 25923)	MIC (%) = 0.020, MBC (%) = 0.05	
	*E. coli* (ATCC 25922)	MIC (%) = 0.025, MBC (%) = 0.05	
	*P. aeruginosa* (ATCC 27853)	MIC (%) = 0.025, MBC (%) = 0.05	
	*S. typhimurium* (ATCC 1402)	MIC (%) = 0.025, MBC (%) = 0.05	
MeOH–H_2_O	*B. cereus* (ATCC10876)	MIC (mg/mL) = 2.34	[24]
	*M. luteus* (NRLL B-4375)	MIC (mg/mL) = 7.03	
	*P. mirabilis* (ATCC35659)	MIC (mg/mL) = 4.68	
	*E. coli* (ATCC25922)	MIC (mg/mL) = 9.37	
	*S. typhimurium* (ATCC13311)	MIC (mg/mL) = 7.06	
n–BuOH	*E. coli* (ATCC25922)	IZ (mm) = 7	[17]
	*P. aeruginosa* (ATCC27853)	IZ (mm) = 6.5 ± 0.7	
	*S. aureus* (ATCC25923)	IZ (mm) = 8	
	*E. faecalis* (ATCC29212)	IZ (mm) = 7	
EtOH & EO	*S. epidermidis* ATCC12228	MIC (*μ*g/mL) = 128 (EtOH), 32 (EO)	[23]
	*S. aureus* ATCC25923	MIC (*μ*g/mL) = 128 (EtOH), 32 (EO)	
	*B. subtilis* ATCC11562	MIC (*μ*g/mL) = 64 (EtOH), 32 (EO)	
	*E. coli* ATCC29425	MIC (*μ*g/mL) = 256 (EtOH), 64 (EO)	
	*P. aeruginosa* ATCC15442	MIC (*μ*g/mL) = 512 (EtOH), 512 (EO)	
	*K. pneumoniae* ATCC43816	MIC (*μ*g/mL) = 256 (EtOH), 256 (EO)	
H_2_O & EO	*P. aeruginosa*	IZ (mm) = 19–55 (H_2_O)	[14]
	*E. coli*	IZ (mm) = 35–44 (EO)	
	*S. aureus*	IZ (mm) = 44–55 (EO)	
	*E. aerogenes*	IZ (mm) = 19–34 (EO)	
MeOH & EtOH	*E. coli* ATCC 25922	IZ (mm) = 13 (MeOH), 10 (EtOH), MIC (*μ*g/mL) = 220 (MeOH), 270 (EtOH)	[70]
	*K. pneumonia* ATCC 4352	IZ (mm) = 0 (MeOH), 0 (EtOH), MIC (*μ*g/mL) = 0 (MeOH), 0 (EtOH)	
	*P. aeruginosa* ATCC 27853	IZ (mm) = 16.5 (MeOH), 14 (EtOH), MIC (*μ*g/mL) = 185 (MeOH), 150 (EtOH)	
	*S. typhimurium* ATCC 13311	IZ (mm) = 9 (MeOH), 12 (EtOH), MIC (*μ*g/mL) = 110 (MeOH), 130 (EtOH)	
	*E. cloacae* ATCC 49452	IZ (mm) = 7 (M), 0 (EtOH), MIC (*μ*g/mL) = 160 (MeOH), 0 (EtOH)	
	*E. faecalis* ATCC 49452	IZ (mm) = 12.5 (MeOH), 17 (EtOH), MIC (*μ*g/mL) = 80 (M), 105 (EtOH)	
	*S. aureus* ATCC 25923	IZ (mm) = 19 (MeOH), 15.5 (EtOH), MIC (*μ*g/mL) = 40 (MeOH), 65 (EtOH)	
EO, EtOH, & H_2_O	*E. coli*	IZ (mm) = 11.53 ± 0.43 (EO), 10.91 ± 0.05 (EtOH)	[72]
	*S. aureus*	IZ (mm) = 11.52 ± 0.41 (EO)	
	*P. aeruginosa*	IZ (mm) = 0 (EO)	
	*S. enterica*	IZ (mm) = 12.51 ± 0.19 (EO)	
EtOH–H_2_O & H_2_O	*M. morganii*	MIC (mg/mL) = 10 (H_2_O)–5 (EtOH–H_2_O)	[7]
	*P. aeruginosa*	MIC (mg/mL) = 20 (H_2_O)–20 (EtOH–H_2_O)	
	*E. coli*	MIC (mg/mL) = 5 (H_2_O)–5 (EtOH–H_2_O)	
	*E. coli* extended producer of *β*–lactamases (ESBL)	MIC (mg/mL) = 5 (H_2_O)–5 (EtOH–H_2_O)	
	*K. pneumoniae*	MIC (mg/mL) = 10 (H_2_O)–5 (EtOH–H_2_O)	
	*K. pneumoniae* extended producer of *β*–lactamases (ESBL)	MIC (mg/mL) = 10 (H_2_O)–5 (EtOH–H_2_O)	
	*E. faecalis*	MIC (mg/mL) = 10 (H_2_O)–10 (EtOH–H_2_O)	
	*L. monocytogenes*	MIC (mg/mL) = 10 (H_2_O)–10 (EtOH–H_2_O)	
	*S. aureus (*MSSA)	MIC (mg/mL) = 5 (H_2_O)–2.5 (H_2_O & H_2_O)	
	*S. aureus* (MRSA)	MIC (mg/mL) = 5 (H_2_O)–2.5 (H_2_O & H_2_O)	
EO	*E. coli* ATCC 25922	IZ (mm) = 28 ± 1.5	[151]
	*S. typhimurium* ATCC 1402	IZ (mm) = 20 ± 1.73	
	*S. aureus* ATCC 25923	IZ (mm) = 12 ± 1.33	
	*P. aeruginosa* ATCC 27853	IZ (mm) = 13 ± 1	
EO	*E. coli*	IZ (mm) = 10–13	[118]
	*S. aureus*	IZ (mm) = 8–36	
	*B. subtilis*	IZ (mm) = 10–13	
	*K. pneumoniae*	IZ (mm) = 0	
H_2_O & MeOH	*S. typhimurium*	MIC (mg/mL) = 0.25–0.5 (H_2_O), 0.12–0.25 (MeOH)	[19]
	*E. coli*	MIC (mg/mL) = 0.12–0.5 (H_2_O), 0.12–0.25 (MeOH)	
	*S. aureus*	MIC (mg/mL) = 0.5–1 (H_2_O), 1 (MeOH)	
	*S. epidermis*	MIC (mg/mL) = 0.12–0.5 (H_2_O), 0.5–1 (MeOH)	
EO	*S. aureus* (ATCC 6538)	MIC (mg/mL) = 0.08 ± 0.03, MBC (mg/mL) = 0.15 ± 0.05	[6]
	*S. typhimurium* (ATCC 13311)	MIC (mg/mL) = 0.09 ± 0.04, MBC (mg/mL) = 0.18 ± 0.07	
	*E. cloacae* (human isolate)	MIC (mg/mL) = 0.05 ± 0.04, MBC (mg/mL) = 0.11 ± 0.07	
	*E. coli* (ATCC 35210)	MIC (mg/mL) = 0.08 ± 0.03, MBC (mg/mL) = 0.11 ± 0.07	
	*P. aeruginosa* (ATCC 27853)	MIC (mg/mL) = 0.05 ± 0.00, MBC (mg/mL) = 0.11 ± 0.01	
	*L. monocytogenes* (NCTC 7973)	MIC (mg/mL) = 0.04 ± 0.00, MBC (mg/mL) = 0.09 ± 0.02	
	*M. flavus* (ATCC 10240)	MIC (mg/mL) = 0.03 ± 0.00, MBC (mg/mL) = 0.05 ± 0.00	
	*B. cereus* (clinical isolate)	MIC (mg/mL) = 0.04 ± 0.01, MBC (mg/mL) = 0.08 ± 0.02	
EO	*E. coli* ATCC 25922	MIC (mg/mL) = 2.5 mg/mL	[136]
	*P. aeruginosa* ATCC 27853	MIC (mg/mL) = 1.66 mg/mL	
	*S. aureus* ATCC 25923	MIC (mg/mL) = 0.20 mg/mL	
EO	*K. pneumoniae*	MIC (mg/mL) = 2.030–2.114, MBC (mg/mL) ≥ 4.227	[67]
	*P. aeruginosa*	MIC (mg/mL) ≥ 4.227, MBC (mg/mL) ≥ 4.227	
	*S. Typhi*	MIC (mg/mL) = 2.114–3.004, MBC (mg/mL) = 4.059–3.044	
	*E. coli*	MIC (mg/mL) = 3.004–3.044, MBC (mg/mL) ≥ 4.059	
	*B. cereus*	MIC (mg/mL) = 0.264–1.015, MBC (mg/mL) = 0.528–1.015	
	*S. aureus*	MIC (mg/mL) = 1.015–1.057, MBC (mg/mL) = 1.015–1.057	
	*S. aureus* (MRSA)	MIC (mg/mL) = 0.528–1.015, MBC (mg/mL) = 2.030–3.044	
	*E. faecalis*	MIC (mg/mL) = 0.507–0.528, MBC (mg/mL) = 1.015–1.057	
EO	*E. coli* GM 109	MIC (mg/mL) = 1.80–4.20	[20]
	*P. aeruginosa*	MIC (mg/mL) = 0.90–0.90	
	*S. enteritidis* ATCC 502	MIC (mg/mL) = 1.50–22.00	
	*S. aureus* ATCC 25923	MIC (mg/mL) = 1.70–4.50	
	*B. subtilis* 166	MIC (mg/mL) = 4.00–5.50	
	*L. monocytogenes*	MIC (mg/mL) = 2.00–7.50	
EO	*S. mutans* (IBR S001)	MIC (*μ*g/mL) = 40 ± 1.15, MBC (*μ*g/mL) = 80 ± 2.25	[73]
	*S. aureus* (ATCC 25923)	MIC (*μ*g/mL) = 80 ± 2.25, MBC (*μ*g/mL) = 160 ± 4.50	
	*S. salivarius* (IBR S006)	MIC (*μ*g/mL) = 40 ± 3.00, MBC (*μ*g/mL) = 80 ± 5.95	
	*S. sanguinis* (IBR S002)	MIC (*μ*g/mL) = 40 ± 0.00, MBC (*μ*g/mL) = 80 ± 0.00	
	*S. pyogenes* (IBR S004)	MIC (*μ*g/mL) = 40 ± 0.00, MBC (*μ*g/mL) = 80 ± 0.00	
	*E. feacalis* (IBR E001)	MIC (*μ*g/mL) = 20 ± 3.40, MBC (*μ*g/mL) = 40 ± 6.75	
	*P. aeruginosa* (IBR P001)	MIC (*μ*g/mL) = 80 ± 2.25, MBC (*μ*g/mL) = 160 ± 4.50	
	*L. acidophilus* (IBR L001)	MIC (*μ*g/mL) = 40 ± 0.00, MBC (*μ*g/mL) = 80 ± 0.00	
EO	*E. coli* (ATCC 35210)	MIC (mg/mL) = 0.002, MBC (mg/mL) = 0.004	[48]
	*P. aeruginosa* (ATCC 27853)	MIC (mg/mL) = 0.003, MBC (mg/mL) = 0.05	
	*S. typhimurium* (ATCC 13311)	MIC (mg/mL) = 0.05, MBC (mg/mL) = 0.05	
	*P. mirabilis* (human isolate)	MIC (mg/mL) = 0.003, MBC (mg/mL) = 0.05	
	*L. monocytogenes* (NCTC 7973)	MIC (mg/mL) = 0.001, MBC (mg/mL) = 0.05	
	*B. cereus* (clinical isolate)	MIC (mg/mL) = 0.001, MBC (mg/mL) = 0.0025	
	*M. flavus* (ATCC 10240)	MIC (mg/mL) = 0.001, MBC (mg/mL) = 0.0025	
	*S. aureus* (ATCC 6538)	MIC (mg/mL) = 0.002, MBC (mg/mL) = 0.003	
EO	*E. coli* O157:H7 VTEC (phage type 34)	Inactivation of 5 log_10_ cycles of *E. coli* O157:H7 at both pH and of *L. monocytogene*s EGD-e at pH 4.	[35]
	*L. monocytogenes* EGD-e		
EO	*S. enteritidis* (CECT 4155)	IZ (mm) = 15.6 ± 2.4	[96]
	*E. coli* O157:H7 (CECT 4267)	IZ (mm) = 17.8 ± 1.7	
	*P. aeruginosa* (CECT 110)	IZ (mm) = 15.2 ± 1.0	
	*S. aureus* (CECT 239)	IZ (mm) = 51.0 ± 3.4	
	*E. aecalis* (CECT 410)	IZ (mm) = 14.7 ± 1.2	
	*L. monocytogenes* 4b (CECT 935)	IZ (mm) = 26.7 ± 2.3	
	*L. monocytogenes* (EGD-e)	IZ (mm) = 33.7 ± 0.4	
EO	*E. coli* ATCC 25922	IZ (mm) = 14 ± 1 mm, MIC (*μ*L/mL) = 6	[28]
	*P. aeruginosa* ATCC 27853	IZ (mm) = 14.5 ± 0.5 mm, MIC (*μ*L/mL) = 6	
	*K. pneumoniae* ATCC 13883	IZ (mm) = 13.5 ± 0.5 mm, MIC (*μ*L/mL) = 6	
	*S. typhimurium* NRRLB 4420	IZ (mm) = 15 ± 0.5 mm, MIC (*μ*L/mL) = 6	
	*B. cereus* ATCC 11778	IZ (mm) = 30 ± 2 mm, MIC (*μ*L/mL) = 1	
	*E. faecalis* ATCC 29212	IZ (mm) = 18.5 ± 0.5 mm, MIC (*μ*L/mL) = 3	
EO	*B. subtilis*	MIC (*v*/*v*) = 1/250	[57]
	*E. coli*	MIC (*v*/*v*) = 1/500	
	*M. luteus*	MIC (*v*/*v*) = 1/500	
	*S. aureus*	MIC (*v*/*v*) = 1/500	
EO	*S. aureus* CFSA2	IZ (mm) = 9.33 mm	[50]
	*L. monocytogenes* EGD	IZ (mm) = 11.66 mm	
	*B. cereus* C1060	IZ (mm) = 17.00 mm	
	*Salmonella* sp.	IZ (mm) = 8.33 mm	
	*H. pylori* strains J99 and 26695	IZ (mm) = 13–30 mm	
EO	*B. subtilis* ATCC 6633	IZ (mm) = 42 mm, MIC (*μ*L/mL) = 0.5	[52]
	*S. aureus* CIP 7625	IZ (mm) = 0 mm, MIC (*μ*L/mL) = 2	
	*E. coli* CIP 54.8	IZ (mm) = 0 mm, MIC (*μ*L/mL) = 5	
	*P. aeruginosa* CIP A22	IZ (mm) = 0 mm, MIC (*μ*L/mL) = 2	
*Leaves*			
H_2_O	*S. aureus* ATCC 25923	Not active towards any of the microorganisms	[89]
	*P. aeruginosa* ATCC 27853		
	*E. coli* ATCC 25922		
	*B. cereus* ATCC 10876		
MeOH	*S. aureus*	MIC (mg/mL) = 1.4	[25]
	*S. faecalis*	MIC (mg/mL) = 1.4	
	*B. cereus*	MIC (mg/mL) = 1.4	
	*S. epidermis*	MIC (mg/mL) = 1.4	
	*P. aeruginosa*	MIC (mg/mL) = 1.4	
	*E. coli*	MIC (mg/mL) = 1.4 mg/mL, MBC (mg/mL) = 1.4	
	*K. pneumonia*	MIC (mg/mL) = 1.4	
EO	*E. coli* ATCC 25.922	MIC (*μ*L/mL) = 3.25–5	[26]
	*P. aeruginosa* ATCC 9027	MIC (*μ*L/mL) = 3.5–5	
	*S. aureus* ATTCC 25.923	MIC (*μ*L/mL) = 1.25–2.5	
	*L. monocytogenes* ATCC 7644	MIC (*μ*L/mL) = 1.75–4.5	
	*B. cereus* ATCC 11.778	MIC (*μ*L/mL) = 1–2.5	
EO	*K. pneumoniae*	IZ (mm) = 25	[46]
	*E. coli*	IZ (mm) = 46	
	*P. aeruginosa*	IZ (mm) = 75	
	*M. luteus*	IZ (mm) = 15	
	*S. aureus*	IZ (mm) = 60	
	*S. epidermidis*	IZ (mm) = 28	
	*B. bronchiseptica*	IZ (mm) = 25	
	*E. faecalis*	IZ (mm) = 49	
*Leaves & flowers*			
EO	*S. typhimurium*	MIC (mg/mL) = 0.5	[27]
	*E. coli*	MIC (mg/mL) = 0.5	
	*S. aureus*	MIC (mg/mL) = 0.5	
	*S. epidermis*	MIC (mg/mL) = 0.5	
*Aerial parts & leaves*			
EO	*E*. *coli* O157:H7 VTEC (phage type 34)	Inactivation of the initial cell populations by 4–5 log_10_ cycles in combination with high hydrostatic pressure	[101]
	*L*. *monocytogenes* EGD-e		

IC_50_: half-maximal inhibitory concentration; IZ: inhibition zone; MBC: minimum bactericidal concentration; MIC: minimum inhibitory concentration; MRSA: meticillin-resistant *Staphylococcus aureus*; MSSA: meticillin-sensitive *Staphylococcus aureus*.

**Table 6:** Antifungal activities of *T. algeriensis* extracts.

Tested strains	Key results	Ref.
Aerial parts		
EO		
*C. tropicalis*	IZ (mm) = 2.04 ± 0.8	[42]
*C. albicans* IPA200	IZ (mm) = 13.0 ± 0.4	
*C. glabrata*	IZ (mm) = 18.0 ± 0.6	
*C. glabrata*	MIC (*μ*L/mL) ≤ 0.5	[75]
*A. fumigates* (human isolate)	MIC (mg/mL) = 0.01 ± 0.00, MFC (mg/mL) = 0.03 ± 0.00	[6]
*A. versicolor* (ATCC 11730)	MIC (mg/mL) = 0.04 ± 0.01, MFC (mg/mL) = 0.04 ± 0.03	
*A. ochraceus* (ATCC 12066)	MIC (mg/mL) = 0.01 ± 0.00, MFC (mg/mL) = 0.03 ± 0.00	
*A. niger* (ATCC 6275)	MIC (mg/mL) = 0.01 ± 0.00, MFC (mg/mL) = 0.01 ± 0.00	
*T. viride* (IAM 5061)	MIC (mg/mL) = 0.01 ± 0.00, MFC (mg/mL) = 0.01 ± 0.00	
*P. funiculosum* (ATCC 36839)	MIC (mg/mL) = 0.01 ± 0.01, MFC (mg/mL) = 0.03 ± 0.02	
*P. ochrochloron* (ATCC 9112)	MIC (mg/mL) = 0.01 ± 0.02, MFC (mg/mL) = 0.03 ± 0.02	
*P. aurantiogriseum* (food isolate)	MIC (mg/mL) = 0.02 ± 0.01, MFC (mg/mL) = 0.04 ± 0.01	
*C. albicans*	MIC (mg/mL) = 4.510–4.697, MFC (mg/mL) ≥ 4.697	[67]
*V. destructor*	EO at 0.5% decreased the rate of infestation and caused a mortality rate of 32.6%	[78]
*P. infestans*	IC_50_ (*μ*L/L) = nondetermined value	[77]
*P. ultimum*	IC_50_ (*μ*L/L) = nondetermined value	
*B. cinerea*	IC_50_ (*μ*L/L) ≥ 300	
*R. solani*	IC_50_ (*μ*L/L) = nondetermined value	
*F. oxysporum* f. sp. *radicis-lycopersici*	IC_50_ (*μ*L/L) ≥ 200	
*C. albicans* ATCC 10231	MIC (*μ*g/mL) = 10 ± 0.2, MFC (*μ*g/mL) = 20 ± 0.6	[73]
*C. tropicalis* ATCC 750	MIC (*μ*g/mL) = 5 ± 0.0, MFC (*μ*g/mL) = 10 ± 0.0	
*A. flavus* (ATCC 9643)	MIC (mg/mL) = 0.002, MFC (mg/mL) = 0.004	[43]
*A. fumigatus* (human isolate)	MIC (mg/mL) = 0.002, MFC (mg/mL) = 0.003	
*A. niger* (ATCC 6275)	MIC (mg/mL) = 0.001, MFC (mg/mL) = 0.003	
*A. ochraceus* (ATCC 12066)	MIC (mg/mL) = 0.001, MFC (mg/mL) = 0.0025	
*P. funiculosum* (ATCC 36839)	MIC (mg/mL) = 0.001, MFC (mg/mL) = 0.002	
*P. ochrochloron* (ATCC 9112)	MIC (mg/mL) = 0.001, MFC (mg/mL) = 0.0025	
*T. viride* (IAM 5061)	MIC (mg/mL) = 0.0005, MFC (mg/mL) = 0.001	
*C. albicans* (human isolate)	MIC (mg/mL) = 0.025, MFC (mg/mL) = 0.05	
*F. solani*	IZ (mm) = 31 ± 1.5, MIC (*μ*L/mL) = 1	[29]
*A. niger*	IZ (mm) = 64 ± 3, MIC (*μ*L/mL) = 2	
*A. niger*	MIC (*v*/*v*) = 1/500	[27]
*P. expansum*	MIC (*v*/*v*) = 1/500	
*P. digitatum*	MIC (*v*/*v*) = 1/500	
*G. trabeum*	MIC (*v*/*v*) = 1/500	[26]
*P. placenta*	MIC (*v*/*v*) = 1/250	
*C. puteana*	MIC (*v*/*v*) = 1/500	
*C. versicolor*	MIC (*v*/*v*) = 1/250	
*C. albicans*	IZ (mm) = 9.66	[35]
*S. aureus* CFSA2	IZ (mm) = 9.33	
*C. albicans*	IZ (mm) = 32, MIC (*μ*L/mL) = 1	[141]
*S. cerevisiae*	IZ (mm) = 46, MIC (*μ*L/mL) = 1	
*M. ramanniamus* NRRL 6606	IZ (mm) = 28, MIC (*μ*L/mL) = 0.5	
*F. oxysporum f. sp. albedinis*	IZ (mm) = 34, MIC (*μ*L/mL) = 1	
EtOH & EO		
*C. glabrata* ATCC22553	MIC (*μ*g/mL) = 128 (EtOH)–32 (EO)	[23]
*C. albicans* ATCC1023	MIC (*μ*g/mL) = 128 (EtOH)–64 (EO)	
H_2_O & MeOH		
*A. flavus*	%inhibition = 5.33 ± 1.15–8 ± 2 (H_2_O), 0–11.33 ± 1.15 (MeOH)	[19]
*A. niger*	%inhibition = 46.03 ± 2.74–63.83 ± 6.88 (H_2_O), 42.53 ± 0.54–75.04 ± 4.12 (MeOH)	
Leaves		
H_2_O		
*C. albicans* ATCC 10231	Not active	[71]
EO		
*C. albicans*	IZ (mm) = 23	[139]
*S. cerevisiae*	IZ (mm) = 47	
H_2_O & EtOH		
*P. megakarya*	EC_50_ = 32.35 ± 2.02 (H_2_O), EC_90_ = 112.55 ± 16.57 (H_2_O)	[76]
	EC_50_ = 1.32 ± 0.4 (EtOH), EC_90_ = 24.97 ± 4.9 (EtOH)	
Leaves & Flowers		
EO		
*A. niger*	Not active	[69]
*A. flavus*	42.86% inhibition	

EC_50_: half maximal effective concentration; EC_90_: 90% maximal effective concentration; IC_50_: half maximal inhibitory concentration; IZ: inhibition zone; MBC: minimum bactericidal concentration; MFC: minimum fungicidal concentration; MIC: minimum inhibitory concentration.

**Table 7:** *In vivo* activities of *T. algeriensis* extracts.

Extract	Doses	Model	Activity	Effects	Ref.
*Algeria*					
Leaves					
80% MeOH	200, 400, and 600 mg/kg	Male rats	Anti-inflammatory activity	Significant mild reduction in edema thickness using 400 mg/kg by up to 30%	[22]
			Leukocyte's recruitment	A dose-dependent reduction in the total leukocyte number at three doses tested by up to 62%	
	200, 400, and 600 mg/kg	Swiss albino mice	Acetic acid-induced vascular permeability	Attenuation of vasodilation and decreased vascular permeability in mice at 400 or 600 mg/kg by 63 and 58%, respectively	
	200 and 400 mg/kg		Antinociceptive activity by hot plate test	A dose-dependent increase in response latency using 200 and 400 mg/kg by up to 200%	
	200 and 400 mg/kg		Antinociceptive activity by acetic acid-induced abdominal writhing	94% reduction of the writhing response using 400 mg/kg	
	200 and 400 mg/kg	Male Wistar rats	Heat hyperalgesia	Restoration of heat response latency when measured at day 14 post chronic constriction injury (CCI) by about 160 and 200% using 200 and 400 mg/kg, respectively	[47]
			Mechanical hyperalgesia (pinprick test)	Increased the withdrawal time of injured hind paw by 8.4- and 6-folds after 7 and 14 days, respectively	
			Acetone drop test (paw cold allodynia)	Decreased cold allodynia score by about 16- and 10-folds after 7 and 14 days using 200 and 400 mg/kg, respectively	
			Paint-brush test (mechanical dynamic allodynia)	Attenuated the dynamic allodynia score when assessed at day 7 by up to 1.75-folds Normalization of dynamic response score by 400 mg/kg dose level when measured at day 14 post surgery	
Aqueous extract	2000 and 5000 mg/kg	Albino Wistar rats	Acute toxicity study for 14 days	No mortality or signs of toxicity and no significant differences in body weight, food consumption, and absolute organ weights between controls and treated animals	[119]

Aerial part					
MeOH–H_2_O	200, 400, and 800 mg/kg	Albino male mice	Safety evaluation	No influence on the levels of AST and ALT A significant reduction in phosphatase alkaline levels by up to 35% using 400 mg/kg No effect on the levels of urea and creatinine	[24]
			Antioxidant activity	Increased the plasma antioxidant levels by 3-folds (22% of inhibition) using 800 mg/kg	
				Increased the iron reducing ability (908 *μ*M FeSO_4_ eq/mL) using 800 mg/kg by 2-folds compared to the nontreated group (405 *μ*M FeSO_4_ eq/mL) Improved CAT activity by 24 to 86% using 200 to 800 mg/kg Increased GSH levels in mice treated with 400 and 800 mg/kg (34 to 45 nmol/mL) compared to those of the nontreated group (30 nmol/mL) Decreased the MDA levels in the plasma of treated groups at 200 and 400 mg/kg by 50 and 63%, respectively	
*Tunisia*					
Aerial part					
EO	180 mg/kg per day dissolved in normal saline	Sprague Dawley rats	Body weight gain, toxicity, and mortality	Body weight gain, no mortality, and no sign of toxicity after 15 days of experiment	[118]
			Assessment of lipid profile	Decreased the MDA levels (743.57 ± 41.12 nmol MDA/mg protein) compared to the control group (3648.47 ± 33.22 nmol MDA/mg protein)	
			Assessment of antioxidant defense enzymes	Prevented the toxicity effect of H_2_O_2_ on the nonenzymatic antioxidant GSH level and the activities of SOD, CAT, GPx, and GST	
			Histopathological examination	No pathological change, normal histoarchitecture, and significant reduction in neuronal damage induced by H_2_O_2_	
			AChE inhibition	Decreased the AChE activity by up to 31.5%	
	150 mg/kg	Adult male Wistar rats	Reproductive organs weights	Weight gain by up to 92%	[121]
			Sperm morphology	No effect on sperm's morphology, counts, and mobility	
			Sperm count and motility		
			Sperm viability	Increased the sperm's viability by up to 46%	
			Histopathological studies	Improvement in morphological abnormalities (amorphous head, hookless head, doublet heads, compact head tail with a cytoplasmic droplet, irregular tail, and coiled tail) of sperms	
			DNA fragmentation analysis using gel electrophoresis	Protection against H_2_O_2_-induced DNA fragmentation in testis	
			Lipid peroxidation	Reduction of the levels of MDA in testicular cells induced by H_2_O_2_ at 1 mmol/L	
			Assessment of nonenzymatic antioxidants	Prevention of the H_2_O_2_-induced alterations in GSH level	
			Protein estimation	Increase in total protein	
	180 mg/kg per day	Sprague Dawley rats	Hepatic and renal functional marker enzymes	Attenuation of the increase in AST and reduction in urea and creatinine levels in H_2_O_2_-treated group	[122]
			Enzymatic antioxidants and lipid profile	Recovered the levels of CAT (up to 150% increase), SOD (up to 233% increase), GST (up to 15.7% increase) and GPx (up to 71.4% increase) activities, and GSH (up to 98% increase)	
			Histopathological examination	No histopathological changes in the liver and kidney Alleviation of the injuries in the glomeruli and proximal tubules (77.7% reduction in damage score)	
			Body and organ weights	Prevention of H_2_O_2_-induced liver, kidney, and weight loss	
	54, 117, and 180 mL/kg	Adult male and female Wistar rats	Histology of gastric lesions	Lesions inhibition mainly at doses of 180 mg/kg for male rats (88%) and between 117 and 180 mg/kg for female rats (96.25 and 98.85%)	[20]
			Assessment of enzymatic and nonenzymatic antioxidants	Increase in SOD, CAT, GPx, and GST activities and GSH content	
			Measurement of mucus production	Increase in the mucus production of gastric mucosa compared to control group	
			Acute toxicity study in rodents	No signs of toxicity and absence of abnormal organic damage to the rats' organs	

AChE: acetylcholine; ALP: alanine transaminase; AST: aspartate transaminase; CAT: catalase; CCI: chronic constriction injury; CDNB: 2,4-dinitrochlorobenzene; GPx: glutathione peroxidase; GSH: glutathione; GST: glutathione S-transferases; MDA: malondialdehyde; SOD: superoxide dismutase. All experiments were done orally.
